# The role of the left ventral occipitotemporal cortex in speech processing—The influence of visual deprivation

**DOI:** 10.3389/fnhum.2023.1228808

**Published:** 2023-12-06

**Authors:** Gabriela Dziȩgiel-Fivet, Joanna Beck, Katarzyna Jednoróg

**Affiliations:** Laboratory of Language Neurobiology, Nencki Institute of Experimental Biology, Polish Academy of Sciences, Warsaw, Poland

**Keywords:** blind, fMRI, language, plasticity, vOT, VWFA

## Abstract

The role of the left ventral occipitotemporal cortex (vOT) in reading is well-established in both sighted and blind readers. Its role in speech processing remains only partially understood. Here, we test the involvement of the left vOT in phonological processing of spoken language in the blind (*N* = 50, age: 6.76–60.32) and in the sighted (*N* = 54, age: 6.79–59.83) by means of whole-brain and region-of-interest (including individually identified) fMRI analyses. We confirm that the left vOT is sensitive to phonological processing (shows greater involvement in rhyming compared to control spoken language task) in both blind and sighted participants. However, in the sighted, the activation was observed only during the rhyming task and in the speech-specific region of the left vOT, pointing to task and modality specificity. In contrast, in the blind group, the left vOT was active during speech processing irrespective of task and in both speech and reading-specific vOT regions. Only in the blind, the left vOT presented a higher degree of sensitivity to phonological processing than other language nodes in the left inferior frontal and superior temporal cortex. Our results suggest a changed development of the left vOT sensitivity to spoken language, resulting from visual deprivation.

## 1 Introduction

The left ventral occipitotemporal cortex (vOT) is known to be an important part of the reading network. Though the exact function of this region remains a subject of debate (Dehaene and Cohen, 2011; Price and Devlin, 2011), it is activated universally during reading by readers of very different scripts (Rueckl et al., 2015). Moreover, the left vOT is activated not only during print reading using vision but also in a similar manner in Braille readers, both blind and sighted, who read tactually (Reich et al., 2011; Siuda-Krzywicka et al., 2016; Raczy et al., 2019). Its sensitivity to written words changes in the course of reading acquisition (Dehaene-Lambertz et al., 2018), and its connectivity with language processing areas probably has a shaping role in this development (Saygin et al., 2016). The impact of visual deprivation on the course of the vOT development remains unknown. Some findings, however, suggest that this region, along with other parts of the occipital cortex, may become a part of the language network independently of the Braille reading acquisition in the blind (Bedny et al., 2015).

In the blind, the left vOT is also active during speech processing. This activation was shown to be related to syntactic processing (Kim et al., 2017), but some studies suggest that it may also be implicated in phonological processing. Arnaud et al. (2013) found a repetition suppression effect during spoken vowel presentation in the vOT, whereas Burton et al. (2003) presented activation in the vOT during the auditory rhyming task. The role of the left vOT in speech processing in the sighted population was also discussed. Though weaker than in the blind, in the sighted the left vOT can be activated during speech processing (Yoncheva et al., 2010; Ludersdorfer et al., 2016; Planton et al., 2019). It was often explained as automatic activation of the orthographic codes stored in the left vOT connected to the successful reading acquisition (Dehaene et al., 2015) and thus less pronounced in illiterate (Dehaene et al., 2010) or poor readers (Blau et al., 2010; Desroches et al., 2010; Dȩbska et al., 2019). Activation of the left vOT during speech processing in the sighted was shown to depend on the task. When the task does not require access to the orthographic representation, a specific deactivation is observed instead (Yoncheva et al., 2010; Ludersdorfer et al., 2016). It was also suggested that the left vOT in the sighted contains separate neuronal populations for written and spoken language processing (Pattamadilok et al., 2019).

Both learning- and deprivation-induced neuroplasticity have an unquestionable influence on the functional organization of the human brain. These influences may interact with each other. The change in the reading modality and lack of visual inputs to the left vOT region may change its function in a significant way. Recently, we have demonstrated that the superior temporal regions are relatively disengaged during Braille reading (Dziȩgiel-Fivet et al., 2021), a finding that was apparent also in the previous studies on the blind (Burton et al., 2002; Gizewski et al., 2003; Kim et al., 2017). In the sighted population, these temporal sites are thought to be engaged in phonological processing during reading (Kovelman et al., 2012) and speech processing and related to the sequential, phonology-based strategy of reading used by beginning readers (Jobard et al., 2003; Martin et al., 2015). Similar findings of decreased activation in temporal regions accompanied by increased activation of the vOT in the blind compared to sighted were observed during sound categorization (Mattioni et al., 2022). The authors found reduced decoding accuracy in the temporal cortex that was concomitant to the enhanced representation of sound categories in the occipital cortex. This effect was specific to the human voice. These results suggest that visual deprivation may trigger a redeployment mechanism, in which a certain type of processing is relocated from the intact to the deprived cortices. This could be the case for phonological processing typically tagging the superior temporal cortex.

This study aimed to test whether the left vOT is involved in the phonological processing of spoken language in the blind and to see if this involvement is different from the one observed in the sighted participants. We predicted that the left vOT will be sensitive to phonological processing showing greater activation during the phonological than control spoken language task in both blind and sighted individuals. However, we expected this sensitivity to be greater in the blind than in the sighted. Furthermore, we explored whether sensitivity to phonology depends on specific areas of the vOT selectively involved in reading or speech processing. Additionally, we compared the left vOT activation to other typical language areas and the primary visual cortex. If in the blind the vOT is part of the language network, it should exhibit the same degree of sensitivity to phonological processing as other language nodes in the left inferior frontal and superior temporal cortex.

## 2 Materials and methods

### 2.1 Participants

Data from 51 blind (31 women, mean age = 23.94, *SD* = 14.11, range 6.76–60.32) and 54 sighted (31 women, mean age = 22.97, *SD* = 13.58, range 6.79–59.83) participants were included in the analyses. Sample sizes in this study are higher than the sample sizes usually found in studies with blind participants (typically 10–20). The blind sample was of convenience—all the blind participants matching the inclusion criteria (early blindness, Braille as a primary script for reading acquisition, no knowledge of print Latin alphabet, and no contraindications for MRI scanning) were tested. The sighted sample was recruited to match the blind in terms of age, sex, handedness, and education level. Handedness was measured using the Edinburgh Handedness Questionnaire translated into Polish. Most of the participants were right-handed (45 blind and 47 sighted), but almost half of the blind participants preferred using their left hand for reading Braille (22 participants). None of the blind participants has ever learned to read the Latin alphabet visually.

None of the participants had any history of neurological illness or brain damage (other than the cause of blindness) and all of the participants declared having normal hearing. All of the anatomical images were assessed by a radiologist and no brain damage was found in any of the participants. Blind participants were congenitally (*N* = 42) or early blind (*N* = 9, from 6 years of age at the latest) due to pathology in or anterior to the optic chiasm (details on the blindness causes and onset can be found under this link: https://osf.io/kzjw2/).

One blind participant was excluded from all analyses due to excessive motion during scanning. Two sighted participants were not included in the whole-brain analyses because of missing data in their individual masks. Thus, the final group sizes in the whole-brain analyses were as follows: blind: 50 participants, sighted: 52 participants, and the final group sizes in the ROI analyses were as follows: blind: 50 participants, sighted: 54 participants.

As the delayed onset of blindness may influence the organization of language processing in the blind (Burton et al., 2002; Bedny et al., 2012), the analyses were repeated excluding the blind participants who were not congenitally blind. The differences in comparison to the results on the complete sample were minor and thus only the complete sample results are presented in the main text. The results of the restricted sample are presented in the Supplementary material. Since the inclusion of a wide age range could potentially limit the interpretation of results, we report separate ROI analyses on the adult participants only in Supplementary material. Again, the differences in comparison to the results on the complete sample were minor and therefore here we report the complete sample results.

### 2.2 Experimental design

#### 2.2.1 fMRI tasks

Two fMRI tasks were used to answer our research questions—a language localizer and a phonological task. The stimuli for the tasks were presented using the Presentation software (Neurobehavioral Systems, Albany, CA). Auditory stimuli were presented via noise-attenuating headphones (NordicNeuroLab), visual stimuli were displayed on an LCD monitor, and tactile stimuli via NeuroDevice Tacti TM Braille display (Debowska et al., 2013).

##### 2.2.1.1 Language localizer

The language localizer was previously described by Dziȩgiel-Fivet et al. (2021). The participants were asked to silently read (sighted participants from the screen and blind participants from the Braille display) and listen to stimuli in three conditions: real words, pronounceable but nonsense pseudowords, and non-linguistic control stimuli (for the visual condition: 3 or 4 hash signs, for the tactile condition 3 or 4 six dot Braille sign, for the auditory condition vocoded speech stimuli). There was no other task than to read and listen to the stimuli. The task was presented in three runs. Each run consisted of 36 blocks−18 auditory and 18 tactile or visual including 6 blocks per condition. Within each block, four different stimuli from the same condition were presented in succession. Auditory and visual stimuli were displayed for 1,000 ms, while tactile stimuli were displayed for 3,000 ms (Veispak et al., 2012; Kim et al., 2017), with a 1,000 ms interstimulus interval. Blocks were separated with 3,000–6,000 ms breaks. For a more detailed description of the localizer task, please refer to Dziȩgiel-Fivet et al. (2021). Here, only the real words and non-linguistic control conditions were considered. The comparison of these two conditions is supposed to delineate language-specific activations, which are not connected to purely sensory perception.

##### 2.2.1.2 Phonological task

During the phonological (rhyming) task, participants were asked to judge whether auditorily presented pairs of words rhyme or not. In the control task, in which phonological processing was minimal (see Section 5), participants had to decide whether they heard the same word twice or whether the word pair consisted of two different words (Kovelman et al., 2012). The yes/no answers (50% correct responses were “yes” and 50% were “no”) were given by pressing a corresponding button. Each task consisted of 20 common word pairs (all one- to two-syllable nouns), presented in blocks of four pairs each. There were 10 rhyming/same pairs and 10 non-rhyming/different pairs. Both the rhyming and the control task included the same stimuli but were presented during separate runs. Words in pairs were separated by 2 s, and after the second word in a pair, there was a 4-s break for an answer.

### 2.3 MRI acquisition

The data were obtained on the 3T Siemens Trio Scanner. The functional images were acquired in a whole-brain echo-planar imaging (EPI) sequence with 12 channel head coil (language localizer: 32 slices, slice-thickness = 4 mm, TR = 2,000 ms, TE = 30 ms, flip angle = 80°, FOV = 220 mm^3^, matrix size = 64 × 64, voxel size: 3.4 × 3.4 × 4 mm; phonological tasks: 35 slices, slice-thickness = 3.5 mm, TR = 2,000 ms, TE = 30 ms, flip angle = 90°, FOV = 224 mm^3^, matrix size = 64 × 64, and voxel size: 3.5 × 3.5 × 3.5 mm). The anatomical images were acquired using T1-weighted (T1w) MPRAGE sequence with 32 channel head coil (176 slices, slice-thickness: 1 mm, TR = 2,530 ms, TE = 3.32 ms, flip angle = 7°, matrix size = 256 × 256, and voxel size = 1 × 1 × 1 mm).

### 2.4 Whole-brain analyses

Preprocessing of the MRI data and whole-brain analyses were conducted using SPM12 (SPM12, Wellcome Trust Centre for Neuroimaging, London, UK) running on Matlab2017b (The Math-Works Inc. Natick, MA, USA). The standard preprocessing pipeline was applied. First, for all of the functional data, the realignment parameters were estimated (realignment to the mean functional image), and the data was slice-time corrected and resliced. The anatomical images were then coregistered to the mean functional image and segmented based on the template provided in SPM. Afterward, the normalization of the functional data to the MNI space was carried out with the voxel size of 2 × 2 × 2 mm. Finally, images were smoothed with an 8 mm isotropic Gaussian kernel. The ART toolbox (https://www.nitrc.org/projects/artifact_detect) was used additionally to create movement regressors as well as to detect the excessive in-scanner motion—movement over 2 mm and rotation over 0.2 mm in relation to the previous volume (default ART toolbox settings). To include a session in the analyses, 80% of the volumes needed to be artifact free. One session of one participant had to be excluded and as it was the control task run for the phonological activity analysis; this participant had to be excluded from all analyses.

Preprocessed data were analyzed using a voxel-wise GLM approach. The condition blocks were convolved with the canonical hemodynamic function, and movement and motion outliers regressors were added to the model. The masking threshold in the first level model specification was defined as 0.5 to ensure good coverage of the temporal and occipitotemporal regions by the individual participants' brain masks.

For detailed results of the localizer task see the Supplementary material. We replicated the main findings of Dziȩgiel-Fivet et al. (2021), i.e., the relative deactivation of the temporal sites during Braille reading and inclusion of the vOT to modality-independent language network in the blind in a larger sample including children.

The second-level analyses were conducted on the phonological task. One-sample *t*-tests were used to delineate regions involved in phonological processing (rhyming > control) within groups and two-sample *t*-tests to show the differences between the groups.

Additionally, in Supplementary material, we present the results of analyses where each task was compared to baseline (i.e., rhyming > baseline and control > baseline). Deactivation in both tasks was also analyzed (i.e., baseline > rhyming and baseline > control), as previous studies suggested that the activation of the vOTs during speech processing in the sighted depends on the task (Yoncheva et al., 2010; Ludersdorfer et al., 2016). If not otherwise specified, whole brain results are reported at *p* < 0.001 voxel-level threshold with FWE *p* < 0.05 cluster-level correction.

### 2.5 ROI analyses

#### 2.5.1 Literature-based ROIs

The literature-based left vOT ROI was created as a sum of two 10 mm radius spheres around two peaks—one from the Lerma-Usabiaga et al.'s (2018) study, the averaged LEX contrast peaks coordinates (−41.54, −57.67, −10.18), and the second from the Kim et al.'s (2017) study, the peak of activation for the auditory words > backward speech contrast averaged between the blind participants (−41, −44, −17). The two spheres were intersected with the Inferior Temporal Gyrus (ITG) and Fusiform Gyrus (FG) masks coming from the AAL3 atlas (Rolls et al., 2020) in order to exclude voxels from the cerebellum. The ROI was created using the MarsBar toolbox (Brett et al., 2002). The resulting ROI consisted of 761 voxels.

In order to compare the pattern of activation of the left vOT to other parts of the language network, three additional literature-based ROIs were defined. First, the primary visual cortex and Broca's region ROIs were extracted from the Anatomy Toolbox (Eickhoff et al., 2005). Additionally, the left superior temporal gyrus (STG) ROI was defined based on the recent meta-analysis of the studies tapping into phonological and semantic processing (Hodgson et al., 2021) as a 10 mm radius sphere around a peak for phonological > semantic activations in the superior temporal cortex (coordinates: −58, −23, 8).

#### 2.5.2 Individual ROIs

As the location of the language-sensitive voxels in the vOT can be highly variable (Saxe et al., 2006), we used an individual ROIs approach. The individual ROIs were defined based on the language localizer task activation within the volume of the search defined as the sum of two 20 mm radius spheres around the same peaks as the literature-based ROI, intersected with the ITG and FG masks (2,658 voxels).

First, we wanted to select the parts of the vOT that are language sensitive, irrespective of the modality, i.e., areas sensitive to reading processing and/or speech processing. To this end, the 50 most activated voxels (with the highest *t*-value) in the volume of search in the speech > non-linguistic control and reading words > non-linguistic control contrasts were marked. Then, the marked voxels from the two contrasts (reading and speech-processing related) were combined to create the individual language ROI (ranging from 60 to 100 voxels; 50 voxels would reflect a complete overlap between the speech and reading-related ROIs and 100 voxels would reflect no overlap between the speech and reading-related ROIs).

In fact, there was little to none overlap between the speech and reading-related voxels in both blind (mean number of overlapping voxels = 8.78, *SD* = 12.04) and sighted (mean number of overlapping voxels = 4.57, *SD* = 7.61) and the groups did not differ in this respect (*W* = 1570.5, *p* = 0.134). The ROIs were not necessarily constructed of contiguous voxels. In the blind group, the voxel presenting the largest interparticipant overlap (15 ROIs overlapping) was located at −40, −46, and −14. In the sighted group, the voxel presenting the largest interparticipant overlap (13 ROIs overlapping) was located at −39, −46, and −16. The location of the individual ROIs is consistent with the results presented in Dziȩgiel-Fivet et al. (2021), where localization of the peaks of activation related to reading was not discernible between the blind and the sighted group.

Next, we defined two ROIs in the left vOT selective either to reading or speech processing. The speech-selective ROIs were defined as voxels activated during speech-processing more than for reading [(speech > non-linguistic control) > (reading > non-linguistic control)] in the localizer task and the reading-selective ROIs were defined as the voxels activated for reading more than for speech-processing [(reading > non-linguistic control) > (speech > non-linguistic control)]. The 50 most activated voxels were selected for each contrast (highest *t*-value).

In-house scripts written in Matlab that used SPM12 functions (the “spm_summarize” function for the extraction of the contrast estimates values) were used to extract ROI data. The contrast estimates for the rhyming > baseline and control > baseline contrast from the phonological tasks were analyzed. Scripts in R (version 4.04, R Core Team, 2023) were used to analyze the ROI data—compare groups and conditions, and conduct correlation analysis with reading skills, phonological awareness, and age (see Supplementary material).

## 3 Results

### 3.1 Behavioral results

Both blind and sighted participants performed rhyming and control tasks near the ceiling level. In the rhyming task, the sighted group achieved 98.67% (*SD* = 2.97%) accuracy on average, and the blind group achieved 99.15% accuracy (*SD* = 4.59%). There was a significant difference between the groups for the rhyming task accuracy, tested with the Mann–Whitney U test (*W* = 1,486, *p* = 0.036). In the control task, the sighted group scored 99.43% (*SD* = 1.88%) on average, whereas the blind group scored 99.15% (*SD* = 2.51%). The difference between the groups was insignificant for the control task (*W* = 1259.5, *p* = 0.632).

The analysis of reaction times (RT) indicated that the control task was significantly easier (evoked shorter reaction times) than the experimental rhyming task. This was the case for both blind (mean RT rhyming = 1.28 s, *SD* RT rhyming = 0.28 s, mean RT control = 1.16 s, *SD* RT control = 0.34 s, *W* = 1,049, *p* < 0.001) and sighted (mean RT rhyming = 1.33 s, *SD* RT rhyming = 0.30 s, mean RT control = 1.13 s, *SD* RT control = 0.27 s, *W* = 1,335, *p* < 0.001). The differences between the groups in reaction times were not significant for either of the tasks (rhyming *W* = 1,159, *p* = 0.444; control *W* = 1,323, *p* = 0.731).

### 3.2 Whole-brain analyses

**Figure 1:**
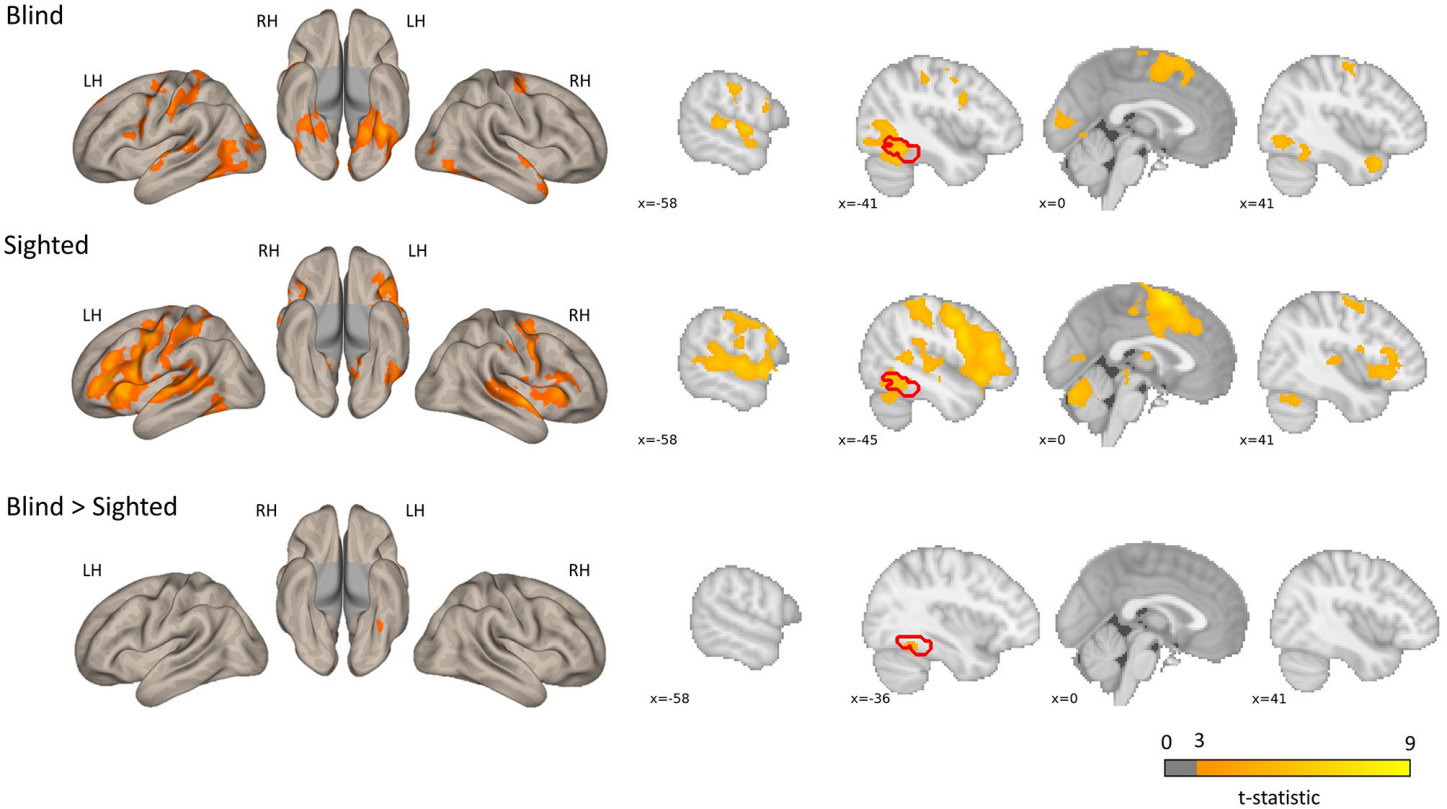
Group level activations and regions activated more by the blind than the sighted for the rhyming > baseline contrast. The blind > sighted contrast did not survive the standard cluster threshold correction (*p* < 0.001 at voxel level, *p* < 0.05 FWE cluster level) and is presented with a more lenient threshold (*p* < 0.001, *k* = 50 voxels). The inverse comparison (sighted > blind) did not yield any significant activations. The left vOT region is marked in red.

The regions sensitive to phonological processing were delineated using contrast comparing the rhyming task to the control task. In this contrast, both groups activated the typical network including perisylvian regions (inferior frontal gyrus, middle temporal gyrus, and superior temporal gyrus), as well as the left vOT (see Figure 1 and Table 1). The blind group additionally activated the primary visual cortex. In the group comparison, a significant difference was found in the left vOT, only when a more lenient statistical threshold (*p* < 0.001, cluster extent 50 voxels, as shown by Bedny et al., 2015) was used—the blind group activated the left vOT cluster to a larger extent than the sighted group. On the statistical threshold used in all other comparisons (*p* < 0.001 on the voxel level, FWE cluster corrected at *p* < 0.05), there were no significant group differences for this contrast.

**Table 1:** Group-level activations and the results of the group comparison of the activations in the rhyming > control contrast.

	**Hem**.	**x**	**y**	**z**	***t***	**vol mm^3^**
**Blind**						
Fusiform gyrus, cerebellum, middle occipital gyrus, lingual gyrus, calcarine, middle temporal gyrus, inferior temporal gyrus, inferior occipital gyrus, parahippocampal gyrus, cuneus, superior occipital gyrus	L	−38	−54	−22	6.09	32,568
Fusiform gyrus, cerebellum, inferior occipital, lingual gyrus, inferior temporal, gyrus, middle occipital gyrus, parahippocampal gyrus	R	36	−42	−18	5.13	10,568
Supplementary motor area, superior frontal gyrus, middle cingulate	R/L	−6	22	62	4.82	9,152
Postcentral gyrus, inferior parietal lobule, superior parietal gyrus, supramarginal gyrus, precuneus, precentral gyrus	L	−54	−20	40	4.88	6,848
Superior temporal gyrus, middle temporal gyrus, heschl gyrus, rolandic operculum, superior temporal pole	L	−58	−8	2	5.71	5,688
Superior and middle temporal pole, superior temporal gyrus, middle temporal gyrus, insula	R	46	16	−28	5.93	4,056
Precentral gyrus, superior frontal gyrus, middle frontal gyrus	R	28	−6	70	4.54	4,016
Precentral gyrus, superior frontal gyrus, middle frontal gyrus	L	−30	−10	62	4.28	3,336
Precentral gyrus, inferior frontal gyrus (pars opercularis, triangularis), rolandic operculum	L	−44	4	30	4.91	3,192
**Sighted**						
Inferior frontal gyrus (pars triangularis, opercularis, orbitalis), precentral gyrus, postcentral gyrus, superior temporal gyrus, supplementary motor area, middle temporal gyrus, insula, middle and anterior cingulate, putamen, inferior parietal lobule, middle frontal gyrus, superior frontal gyrus, superior temporal pole, superior parietal gyrus, rolandic operculum, hippocampus, lingual gyrus, supramarginal gyrus, pallidum, amygdala, heschl gyrus, anterior cingulate, parahippocampal gyrus, thalamus, caudate, cerebellum, precuneus, olfactory cortex, thalamus, fusiform gyrus	R/L	−4	4	60	8.67	140,144
Superior temporal gyrus, precentral gyrus, inferior frontal gyrus (pars triangularis, opercularis, orbitalis), insula, superior and middle temporal pole, superior frontal gyrus, postcentral gyrus, middle frontal gyrus, heschl gyrus, middle temporal gyrus, rolandic opercularis, supramarginal gyrus, putamen	R	62	−6	−6	6.12	41,792
Cerebellum, calcarine, lingual gyrus	R/L	28	−64	−26	5.92	23,192
Inferior temporal gyrus, cerebellum, fusiform gyrus, Inferior occipital gyrus, middle temporal gyrus	L	−48	−60	−12	4.96	4,768
Putamen, caudate	R	16	10	0	5.23	4,336
**Blind** > **Sighted (*****p***<**0.001** ***k*** = **50)**						
Fusiform gyrus	L	−34	−50	−16	3.92	656

The anatomical structures described according to the AAL2 using the “atlasreader” function, Hem., hemisphere; x, y, z, peak coordinates; t, peak t-value; and vol mm^3^.

When each condition was compared to baseline, the suprathreshold activation in the left vOT (and other parts of the occipital cortex) was observed only in the blind group in both tasks (Supplementary Figure 5 and Supplementary Table 6 for rhyming and Supplementary Figure 6 and Supplementary Table 7 for control), and this activation was larger than in the sighted group. Both blind and sighted participants showed deactivation mainly in regions that are a part of the default mode network (anterior, middle, and posterior cingulate, angular gyrus, precuneus, medial frontal cortex; Supplementary Figure 7 and Supplementary Table 8). However, in the sighted group, occipital regions were largely deactivated too. During the control task, the deactivation included bilateral vOT regions. This indicates that the relatively increased activation in the sighted vOT in the rhyming compared to the control task may be a consequence of deactivation in the control task.

### 3.3 ROI analyses

First, we examined the effect of group (blind vs. sighted) and task (rhyming vs. control task) in the literature-based and individual language-sensitive left vOT ROIs. Next, we examined these effects in speech and reading selective left vOT ROIs. Additionally, in order to control for the deactivation effects in the vOT, the activations within the ROIs were compared to zero in every group using the non-parametric one-sample Wilcox signed-rank test. Finally, we compared the activation of the left vOT to other literature-based ROIs (V1, IFG, and STG).

#### 3.3.1 Literature-based and individual language-sensitive left vOT ROIs

As the assumptions for the parametric methods (multilevel modeling ANOVA, using the “lme” function from the “nlme” package, model residuals were not independent of the fitted values) were not met, a robust ANOVA method (“bwtrim” function from the “WRS2” package) was used.

**Figure 2:**
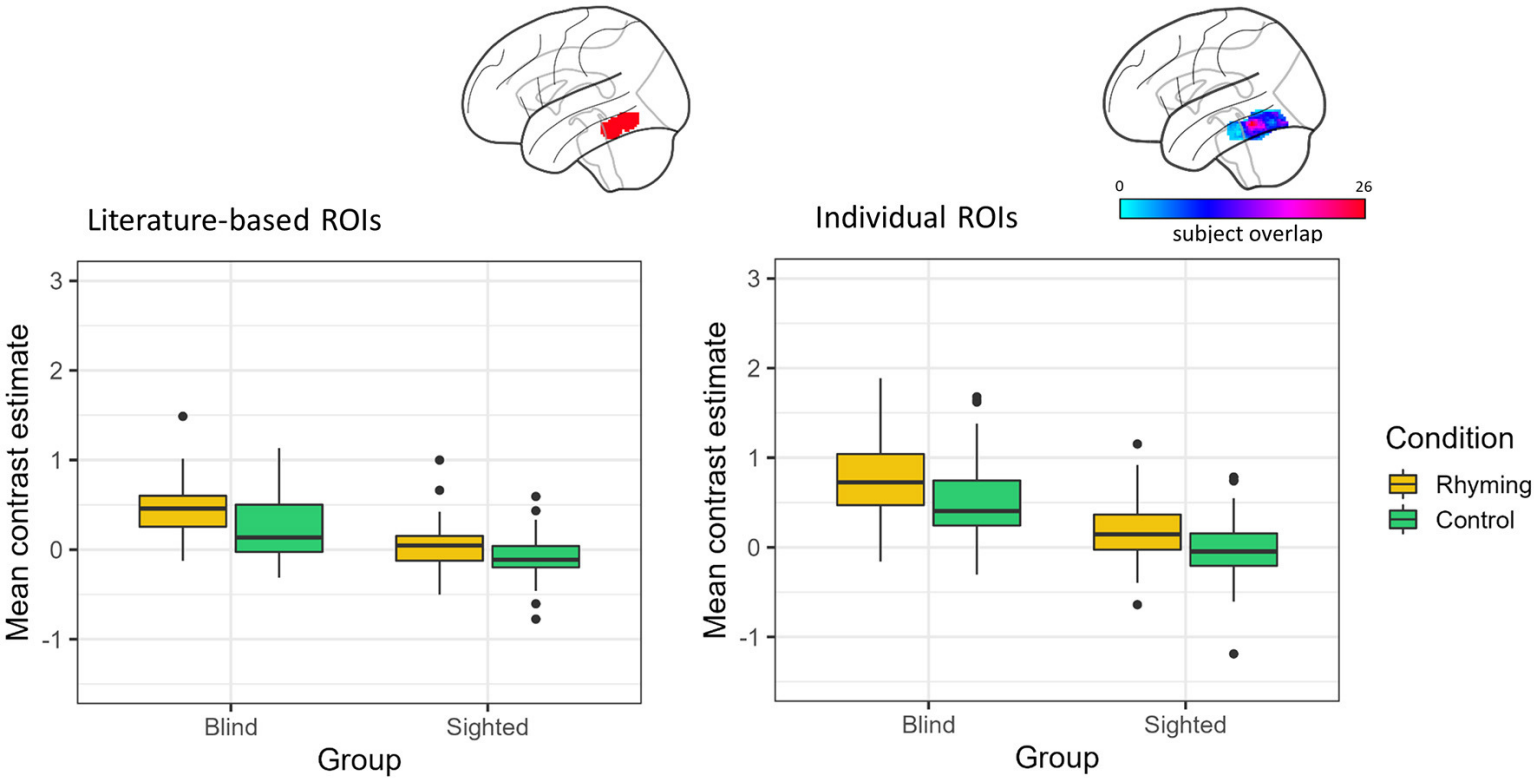
Contrast estimates extracted from the literature-based ROI (its location presented on the brain image) and individual ROIs (color bar depicts the overlap of the ROIs between participants) for the experimental conditions in both groups.

When the literature-based ROI data were analyzed, there was a significant main effect of group [*F*_(1, 45.98)_ = 47.47, *p* < 0.001] with the blind group showing higher activation compared to the sighted group, as well as a significant main effect of condition [*F*_(1, 48.83)_ = 42.39, *p* < 0.001] with rhyming task evoking higher activation than the control task. The interaction of group and condition did not reach significance [*F*_(1, 48.83)_ = 3.92, *p* = 0.053, Figure 2].

Analyses of the data from the ROIs defined individually gave similar results. There was a significant effect of group [*F*_(1, 46.28)_ = 61.16, *p* < 0.001] and condition [*F*_(1, 57.94)_ = 41.02, *p* < 0.001]. The interaction of group and condition was insignificant [*F*_(1, 57.94)_ = 2.36, *p* = 0.130, Figure 2].

The vOT activations were significantly greater than zero for both conditions in the blind group, independently of the ROI type (literature-based ROI: rhyming task mean contrast estimates = 0.45, *SD* = 0.31, *W* = 1,258, *p* < 0.001, control task mean contrast estimates = 0.23, *SD* = 0.35, *W* = 1,030, *p* < 0.001; individual ROIs: rhyming task mean contrast estimates = 0.81, *SD* = 0.55, *W* = 1,266, *p* < 0.001, control task mean contrast estimates = 0.52, *SD* = 0.47, *W* = 1,234, *p* < 0.001). On the other hand, in the sighted the vOT activation was significantly greater than zero only when the rhyming task activation in the individually defined ROIs was taken into consideration (mean contrast estimates = 0.15, *SD* = 0.37, *W* = 1,081, *p* = 0.007). The activity during the control task was significantly below zero when the literature-based ROI data were analyzed (mean contrast estimates = −0.09, *SD* = 0.24, *W* = 395, *p* = 0.006). The rhyming task-related activity in the literature-based ROI (mean contrast estimates = 0.05, *SD* = 0.26, *W* = 879, *p* = 0.242) and the control task-related activity in the individually defined ROIs (mean contrast estimates = −0.04, *SD* = 0.35, *W* = 640, *p* = 0.380) were not significantly different from zero in the sighted group. The deactivation pattern observed in the whole-brain analysis was thus largely confirmed, with the vOT deactivation being task-dependent only in the sighted group. The blind group activated the vOT for speech processing for both rhyming and control conditions.

#### 3.3.2 Reading and speech-selective left vOT

**Figure 3:**
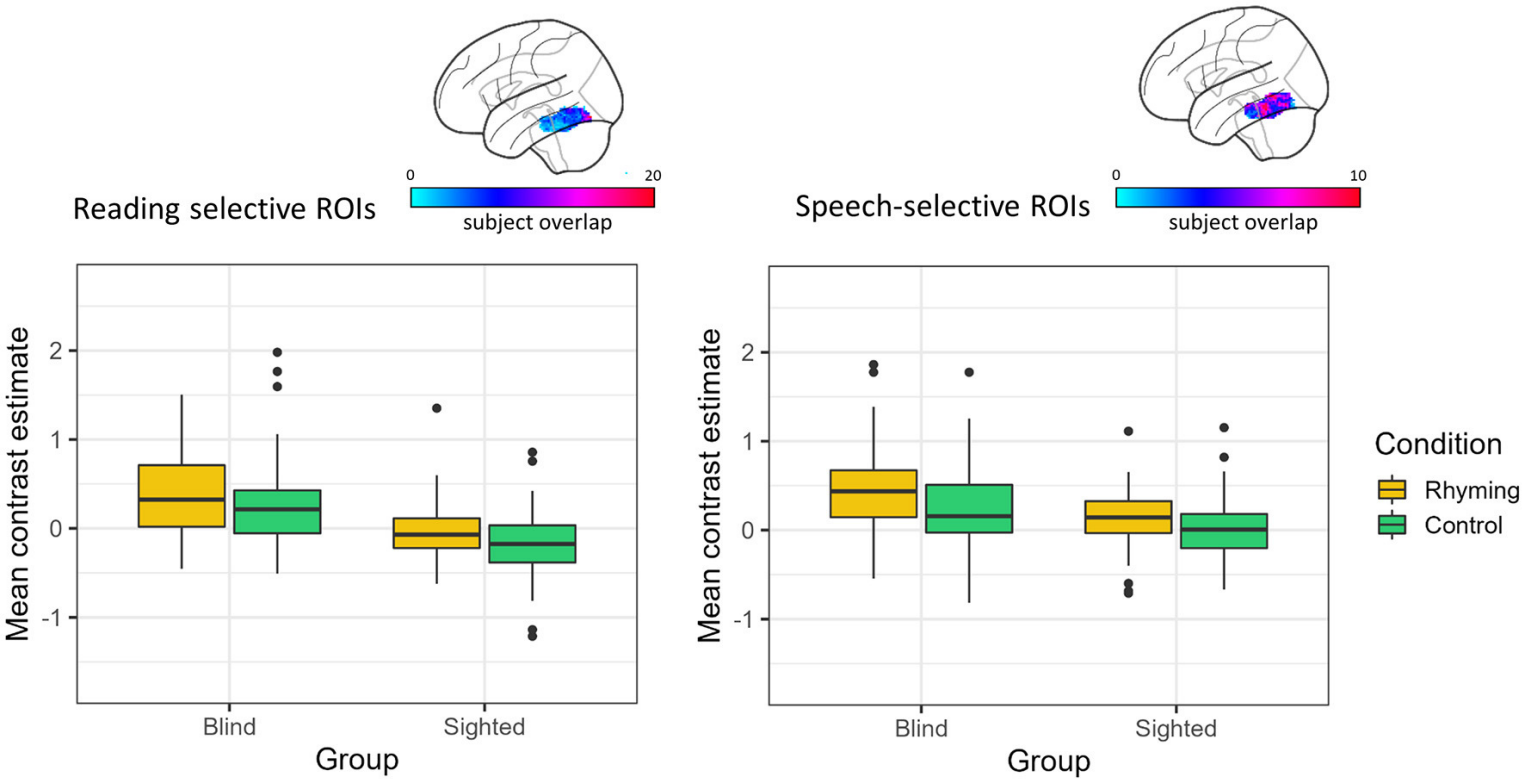
Contrast estimates extracted from the reading and speech-selective individual ROIs. The color bar represents the overlap of the individual ROIs.

The effect of group and condition was analyzed using two-way robust ANOVA (“bwtrim” function) within either speech or reading-selective ROI. In both reading- and speech-selective ROIs, there was a significant main effect of group [reading: *F*_(1, 48.54)_ = 35.01, *p* < 0.001, speech: *F*_(1, 48.16)_ = 13.42, *p* < 0.001; Figure 3] and condition [reading: *F*_(1, 572.95)_ = 13.68, *p* < 0.001, speech: *F*_(1, 57.62)_ = 23.70, *p* < 0.001]. The interaction effect was not significant either in the reading-specific ROIs [*F*_(1, 52.95)_ = 0.09, *p* = 0.249] or in the speech-selective ROIs [*F*_(1, 57.62)_ = 1.39, *p* = 0.244].

For both tasks, the blind group's activations were greater than zero (rhyming: reading: *V* = 1,150, *p* < 0.001, speech: *V* = 1,180, *p* < 0.001; control: reading: *V* = 970, *p* = 0.001, speech: *V* = 1,002, *p* < 0.001). Thus, across tasks and ROI definitions, the blind group activated the left vOT for speech processing (and to a greater extent than the sighted group, see Figure 3).

In the sighted group, the mean contrast estimates were greater than zero only when the rhyming task activations in the speech-selective ROIs were analyzed (*V* = 1,050, *p* = 0.008, Figure 3). The one-sample Wilcox signed-rank tests were insignificant for the control task in the speech-selective ROIs (*V* = 779, *p* = 0.757) and for the rhyming task extracted from the reading-selective ROIs (rhyming: *V* = 578, *p* = 0.158). When the control task activations in the reading-selective ROIs were analyzed, they turned out to be significantly lower than zero (*V* = 344, *p* = 0.001).

#### 3.3.3 Comparison to the other language-network ROIs

Activation of the left vOT was compared to the brain response in V1, STG and Broca's area using a three-way mixed ANOVA was conducted with ROI (vOT vs. V1 vs. STG vs. Broca's) and condition (rhyming vs. control) as the within- participants and group (Blind vs. Sighted) as between- participants factors. The residuals homoscedasticity assumption was not met; however, there are no robust methods for three-way ANOVA, so a classical three-way ANOVA (“anovaRM” function from Jamovi statistical package) was used nevertheless. As the assumption of sphericity was also violated, Greenhouse–Gaiser correction was applied.

**Figure 4:**
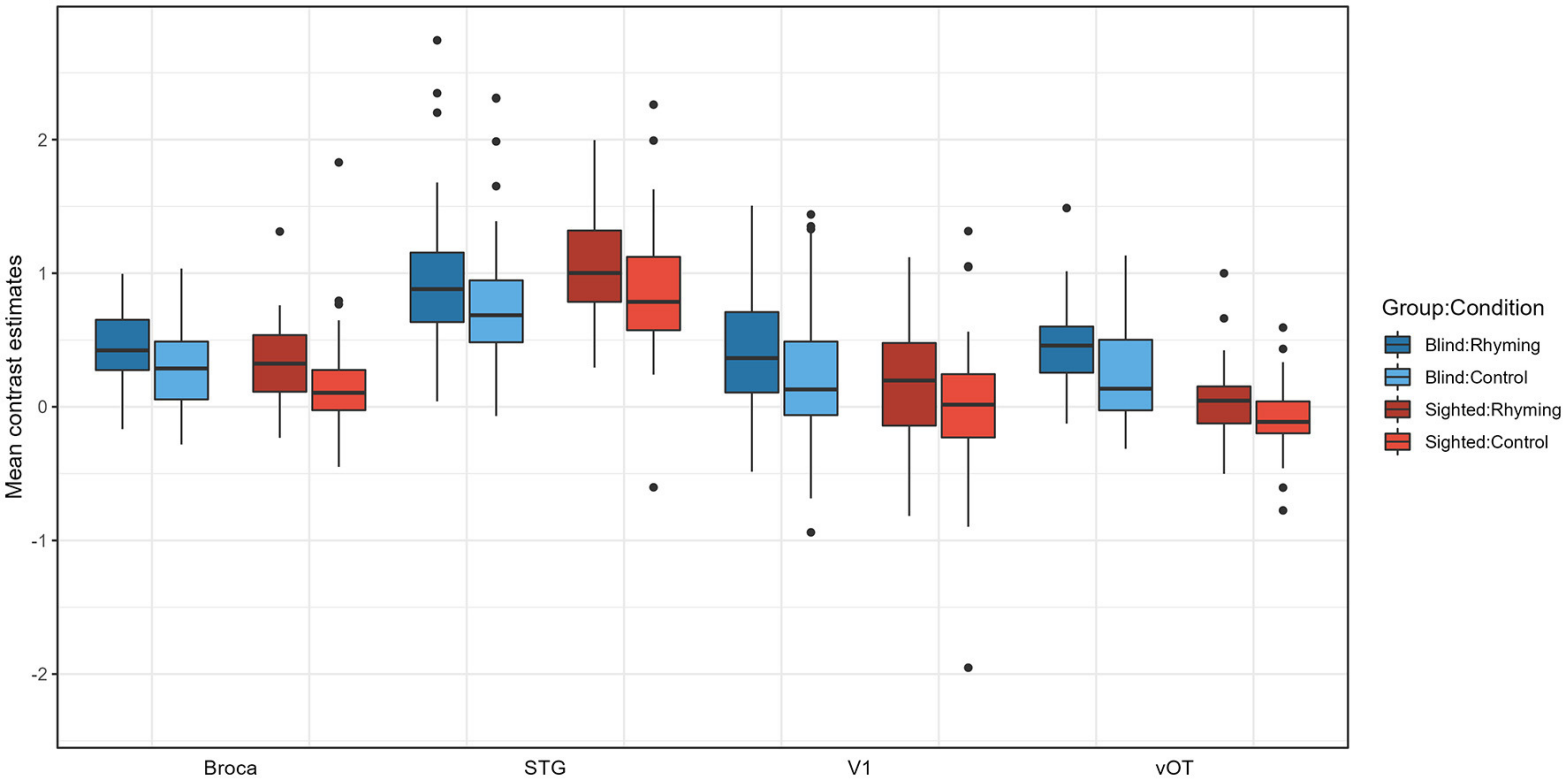
Contrast estimates extracted from the four ROIs for experimental conditions for both groups.

There was a significant main effect of group [*F*_(1, 102)_ = 9.70 *p* = 0.002], ROI [*F*_(2.39, 243.83)_ = 183.11 *p* < 0.001] and condition [*F*_(1, 102)_ = 34.84 *p* < 0.001], as well as significant group × ROI interaction [*F*_(2.39, 243.83)_ = 12.74 *p* < 0.001, Figure 4]. The condition × ROI interaction [*F*_(2.39, 244.20)_ = 0.17 *p* = 0.879], group × condition interaction [*F*_(1, 102)_ = 0.00 *p* = 0.965], and the three-way group × ROI × condition interaction [*F*_(2, 244.20)_ = 1.05 *p* = 0.361] were not significant. The significance of the main effect of group and ROI and the group × ROI interaction was confirmed for both rhyming and control tasks by the robust two-way ANOVA (“bwtrim” function from the “WRS2” package) conducted within conditions.

Pairwise comparisons were conducted (with default Tukey adjustment of *p*-value and *p* < 0.05 significance threshold). *Post-hoc* tests have shown that the activations for rhyming were higher than for the control task in both groups, for vOT but not for other ROIs in the blind (vOT: *p* < 0.001, V1: *p* = 0.368, STG: *p* = 0.107; Broca's area: *p* = 0.252) and for the Broca's, STG, and marginally vOT ROI in the sighted (vOT: *p* = 0.052, V1: *p* = 0.295, STG: *p* = 0.010, Broca's area: *p* = 0.027). Group by ROI interaction can be interpreted as stemming from the fact that in the vOT ROI (Rhyming: *p* < 0.001, Control: *p* < 0.001), for both conditions, activation was higher in the blind group than in the sighted group and the differences between the groups were not significant for the Broca's area (Rhyming: *p* = 0.814, Control *p* = 0.622), V1 (Rhyming: *p* = 0.420, Control *p* = 0.686), and STG ROI (Rhyming: *p* = 1.000, Control *p* = 1.00). In the blind group, for both conditions, STG ROI activation was higher than the three other ROIs (*p*-values of all comparisons < 0.001), and the differences between the Broca's area, V1, and vOT were insignificant (*p*-values of all comparisons >0.889). On the other hand, in the sighted group, for both conditions, not only did STG ROI have higher activation than the three other ROIs (*p*-values of all comparisons < 0.001) but also the Broca's area had higher activation than the vOT ROI (*p* < 0.001). The differences between the vOT and V1, as well as V1 and Broca's area, were not significant (*p*-values of all comparisons >0.242).

## 4 Discussion

This study aimed to test the involvement of the left vOT in phonological processing in both blind and sighted participants. We confirmed that the left vOT is sensitive to phonological processing in both groups, showing increased activation during the rhyming task as compared to the control task (which was also based on linguistic stimuli but should evoke only minimal phonological processing). At the same time, the blind group engaged the left vOT to a greater extent during both tasks than the sighted group. These results resemble the pattern obtained by Kim et al. (2017), where the left vOT in sighted participants responded more to auditory words than backward speech, albeit to a lesser degree than in the blind group. Higher activation for both conditions in the blind group, together with the absence of interaction between group and condition, suggests that the blind vOT is more responsive to phonology than the sighted vOT, but the response profiles do not significantly differ between the groups. Interestingly, in the blind group, the left vOT was activated above baseline during the control task. This contrasted with the sighted participants, where we found left vOT deactivation during the control task, which was consistent with previous findings (Yoncheva et al., 2010; Ludersdorfer et al., 2016; Planton et al., 2019). Only during the rhyming task and specifically within the neuronal populations specialized in processing spoken language, activation in the left vOT was observed in the sighted. This pattern of results could potentially be explained by the presence of phonological groups of neurons within the left vOT that are specifically attuned to phonological features of stimuli (Pattamadilok et al., 2019; for a review of alternative hypotheses, see Dȩbska et al., 2023). In cases where phonological processing is not task relevant, auditory stimuli induced a general deactivation of the visual cortex, including the vOT, relative to rest in the sighted group.

There were no significant differences between the blind and the sighted groups when typical language regions were considered (STG, Broca's area). Yet, only in the blind group, the left vOT presented a higher degree of sensitivity to phonological processing than other language-network nodes. In the sighted, both STG and Broca's area showed stronger activation than the vOT. Interestingly, there was no difference between the activation of V1 and Broca's area in the sighted group. These results suggest that following visual deprivation, vOT becomes a regular node of the language network (engaged in language processing similarly to STG and Broca's region) and is recruited in language processing independently of task demands.

The literature-based ROI used in the current study was quite large and spanned portions of the vOT that may have diverse functional roles (Cohen et al., 2004; Pammer et al., 2004; Vinckier et al., 2007; Bouhali et al., 2019; Ludersdorfer et al., 2019). Additionally, the significant group differences peaked anteriorly to the classical localization of print-sensitive vOT (y = −48 for rhyming > baseline, y = −50 for rhyming > control, compared to y = −57/−58 reported by Cohen et al., 2000; Lerma-Usabiaga et al., 2018). In the sighted, a gradient of specialization was observed with the more anterior parts of the vOT engaged in processing the increasingly complex stimuli with lexical content (Vinckier et al., 2007). The observed group differences may not be bound to the part of the vOT that encompasses orthographic representations in the sighted, but rather to the part of the vOT connected to the semantic system. However, the results of the literature-based ROIs and individually localized ROIs that tapped into the parts of the vOT specifically engaged in reading were the same. The observed pattern of activations was thus present in the area functionally connected to reading. Additionally, the gradient of specialization in the vOT was recently shown to be absent in blind Braille readers (Tian et al., 2023). Current results point to a changed role of the left vOT in the language system of blind individuals.

Although our data do not permit testing this hypothesis directly, we think that the observed differences between the blind and the sighted in the activation during a phonological task reflect a different developmental trajectory in these two groups. The left vOT region is connected to the perisylvian language areas as well as to the occipital cortex (Yeatman et al., 2013). In the sighted population, this unique set of connections is thought to define its crucial role in reading (Dehaene et al., 2015; Saygin et al., 2016). As the left vOT is connected to both visual and linguistic areas it is a perfect candidate for a region binding the newly learned written form of language with the known spoken form. This association is so strong that the left vOT may present some sensitivity to spoken language too (Planton et al., 2019).

In individuals who are congenitally or early blind, the connections of the left vOT probably stay largely unchanged (Noppeney, 2007); however, the nature of the input from the connected areas is different (Bedny, 2017). We know that the language network in the blind is very similar to the one observed in the sighted population, the difference being the inclusion of the occipital cortex (Röder et al., 2002; Dziȩgiel-Fivet et al., 2021). The occipital cortex in the blind is thought to be involved in many high-order cognitive processes and language is one of them (Bedny et al., 2011, 2015). Thus, the left vOT, along with other occipital areas like V1, might also be incorporated into the language processing network, even before Braille reading acquisition. When blind individuals learn how to read, the left vOT becomes active during tactile reading but this activation may reflect more general linguistic processes and not solely the activation of the orthographic representations (Tian et al., 2023).

## 5 Limitations

The rhyming task used was quite easy for the participants, as demonstrated by the analysis of the performance data. The choice of such an easy task was dictated by the large age range of the participants of the study. The task must have been possible to be completed by the minor participants. Second, the control task was chosen based on previous studies focused on phonological processing (Kovelman et al., 2012; Raschle et al., 2014; Yu et al., 2018). However, it consisted of linguistic stimuli and some phonologically related activation, while imminently low-level may have also been evoked by the control stimuli. Finally, it could be the case that our design based on the comparison of rhyming to the control task did not strictly isolate phonological processing but additionally included other linguistic (e.g., semantics) or attention-related processes. Therefore, future studies should use other, more sensitive contrasts to isolate phonology. Finally, the age range in the current study was wider than usually encountered in this type of study. We decided to include such a wide range of participants to maximize the size of the blind sample and thus increase the power of the analyses. Nevertheless, only a few participants at the beginning of reading acquisition were included in the study which prevented us from studying the developmental changes in the vOT sensitivity to language.

## 6 Conclusion

To conclude, we suggest that phonological sensitivity for spoken language in the left vOT in blind participants is different in nature from the one observed in the sighted population. Blind participants activated the left vOT during both rhyming and control spoken language tasks and across the ROI definition methods. Contrarily, the sighted group activation for speech processing was present only in the rhyming task and only within the left vOT neuronal populations specialized in processing spoken language. We hypothesize that in the sighted the sensitivity to spoken language in the left vOT is secondary to its involvement in reading whereas in the blind the sensitivity to speech in this region comes first. Further longitudinal studies are needed to confirm the proposed developmental account of the left vOT response to spoken language in relation to reading acquisition in the sighted and blind populations.

## Data availability statement

The second-level data from the phonological task, participants' demographic characteristics, contrast estimates extracted for the ROI analyses, and scripts used for the ROI analyses can be found at: https://osf.io/kzjw2/.

## Ethics statement

The study was approved by the Scientific Studies Ethics Committee of the Institute of Psychology, Jagiellonian University, which applies The Declaration of Helsinki rules. Adult participants signed an informed consent form at the beginning of the experimental session. The form was sent to the blind participants beforehand in a format readable by the screen reading software. The consent form was signed by the parents of non-adult participants and the verbal consent of children was acquired.

## Author contributions

GD-F: Conceptualization, Data curation, Formal analysis, Investigation, Project administration, Visualization, Writing—original draft, Writing—review & editing. JB: Conceptualization, Data curation, Investigation, Methodology, Project administration, Writing—review & editing. KJ: Conceptualization, Data curation, Funding acquisition, Project administration, Methodology, Supervision, Writing—review & editing.
