# Supplementary material for: The role of the left ventral occipitotemporal cortex in speech processing—The influence of visual deprivation
Source: Front Hum Neurosci. 2023 Dec 6;17:1228808. doi: 10.3389/fnhum.2023.1228808 (PMC10730934; doi:10.3389/fnhum.2023.1228808)
Supplement: Supplementary file 1 [file Data_Sheet_1.docx]

**Supplementary Materials**

Whole-brain analyses

Localizer

Reading

Reading-sensitive activations (words > baseline) were present in the areas typically associated with the reading network (bilateral: vOT, precentral/postcentral gyri, IFG) in both the sighted and the blind (Table S1, Figure S1). For this contrast, the blind subjects activated bilateral occipital clusters (loci of V1/V2) as well as bilateral postcentral gyri and inferior parietal lobule more than the sighted group (Table S1, Figure S2). The sighted subjects activated bilateral temporal cortices (STG, MTG) and bilateral occipital clusters (inferior occipital gyri) more than the blind subjects (Table S1, Figure S2).

Table S1. Group level activations and the results of the group comparison of the activations in the reading words > baseline contrast. The anatomical structures described according to the AAL2 using the “atlasreader” function, Hem. hemisphere, x, y, z – peak coordinates, *t* – peak *t*-value, vol mm^3^ – cluster volume.

|  | Hem. | x | y | z | *t* | vol mm^3^ |
| --- | --- | --- | --- | --- | --- | --- |
| **Blind** |  |  |  |  |  |  |
| Middle Occipital Gyrus, Fusiform Gyrus, Inferior Occipital Gyrus, Middle Temporal, Cerebellum, Superior Occipital Gyrus. Inferior Temporal Gyrus, Calcarine, Lingual Gyrus | L | -42 | -68 | -6 | 8.2 | 28192 |
| Precentral Gyrus, Inferior Frontal Gyrus (pars opercularis, triangularis, orbitalis) | L | -52 | 4 | 38 | 8.13 | 14632 |
| Postcentral Gyrus, Inferior Parietal Lobule, Superior Parietal Lobule, Supramarginal Gyrus | R | 48 | -24 | 40 | 7.14 | 12848 |
| Postcentral Gyrus, Inferior Parietal Lobule, Superior Parietal Lobule, Supramarginal Gyrus | L | -46 | -32 | 44 | 7.28 | 12032 |
| Cerebellum, Inferior Temporal Gyrus, Fusiform Gyrus, Inferior Occipital Gyrus | R | 30 | -64 | -24 | 6.84 | 9384 |
| Precentral Gyrus, Inferior Frontal Gyrus (pars opercularis) | R | 54 | 8 | 26 | 7.09 | 8136 |
| Middle Occipital Gyrus, Superior Occipital Gyrus, Cuneus, Calcarine | R | 30 | -90 | 10 | 4.47 | 4936 |
| Supplementary Motor Area | L/R | -4 | -2 | 64 | 6.43 | 4200 |
| **Sighted** |  |  |  |  |  |  |
| Middle Occipital Gyrus, Inferior Occipital Gyrus, Fusiform Gyrus, Cerebellum, Inferior Temporal Gyrus, Lingual Gyrus, Calcarine, Superior Occipital Gyrus | L | -28 | -92 | -4 | 16.87 | 29976 |
| Inferior Occipital Gyrus, Middle Occipital Gyrus, Inferior Temporal Gyrus, Fusiform Gyrus, Lingual Gyrus, Cerebellum, Calcarine, Cuneus, Superior Occipital Gyrus, Middle Temporal Gyrus | R | 24 | -96 | 6 | 17.23 | 23224 |
| Superior Temporal Gyrus, Middle Temporal Gyrus, Rolandic Operculum, Heschl Gyrus, Postcentral Gyrus | L | -62 | -20 | 2 | 8.29 | 21360 |
| Superior Temporal Gyrus, Middle Temporal Gyrus, Heschl Gyrus, Rolandic Operculum | R | 62 | -30 | 6 | 7.7 | 16840 |
| Precentral Gyrus, Inferior Frontal Gyrus (pars triangularis, opercularis), Postcentral Gyrus | L | -38 | 2 | 30 | 6.99 | 11056 |
| Precentral Gyrus, Inferior Frontal Gyrus (pars opercularis) | R | 44 | 8 | 34 | 5.07 | 2504 |
| **Blind > Sighted** |  |  |  |  |  |  |
| Lingual Gyri, Calcarine, Cuneus, Superior Occipital Gyrus, Cerebellum, Fusiform Gyri, Middle Occipital Gyri | L/R | 2 | -84 | 14 | 9.69 | 59248 |
| Postcentral Gyrus, Superior Parietal Lobule, Supramarginal Gyrus, Inferior Parietal Lobule | R | 48 | -22 | 38 | 8.26 | 16616 |
| Postcentral Gyrus, Inferior Parietal Lobule, Superior Parietal Lobule, Supramarginal Gyrus | L | -50 | -28 | 42 | 7.08 | 14088 |
| Inferior Frontal Gyrus (pars opercularis), Precentral Gyrus | R | 54 | 8 | 24 | 5.37 | 2160 |
| **Sighted > Blind** |  |  |  |  |  |  |
| Precuneus, Middle Cingulate, Angular Gyrus, Supplementary Motor Area, Posterior Cingulate, Middle Occipital Gyrus, Paracentral Lobule, Middle Temporal Gyrus, Cuneus, Inferior Parietal Lobule | L/R | 4 | -30 | 36 | 5.89 | 43024 |
| Superior Temporal Gyrus, Insula, Heschl Gyrus, Rolandic Operculum, Hippocampus, Middle Temporal Gyrus | R | 46 | -20 | 8 | 7.3 | 22696 |
| Superior Temporal Gyrus, Middle Temporal Gyrus, Insula, Heschl Gyrus, Hippocampus, Rolandic Operculum | L | -38 | -32 | 14 | 7.26 | 21688 |
| Middle Occipital Gyrus, Inferior Occipital Gyrus, Calcarine, Superior Occipital Gyrus, Lingual Gyrus, Fusiform Gyrus | L | -22 | -92 | -2 | 10.09 | 6392 |
| Inferior Occipital Gyrus, Middle Occipital Gyrus | R | 24 | -90 | 0 | 8.68 | 6032 |
| Angular Gyrus, Middle Occipital Gyrus | L | -34 | -68 | 28 | 4.83 | 5720 |
| Superior Frontal Gyrus, Middle Frontal Gyrus | R | 24 | 14 | 50 | 4.43 | 2312 |


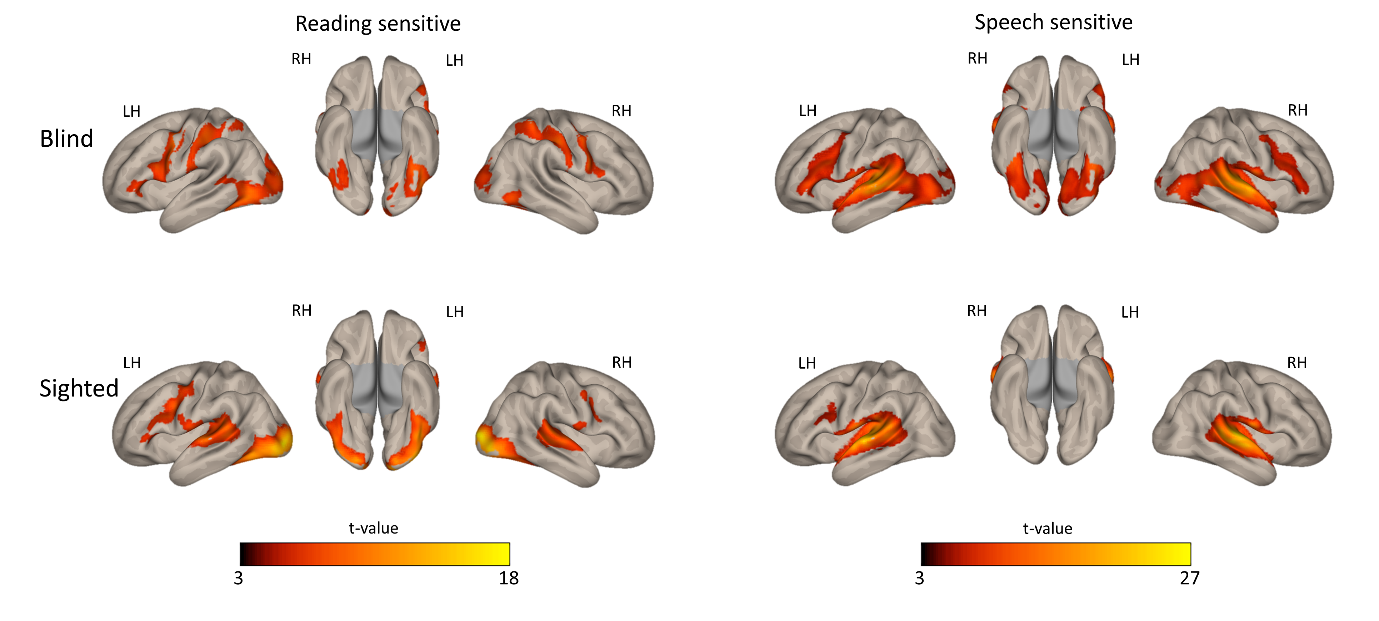


Figure S1. Group level activations for the language-sensitive contrasts (words > baseline) during both reading and speech processing.


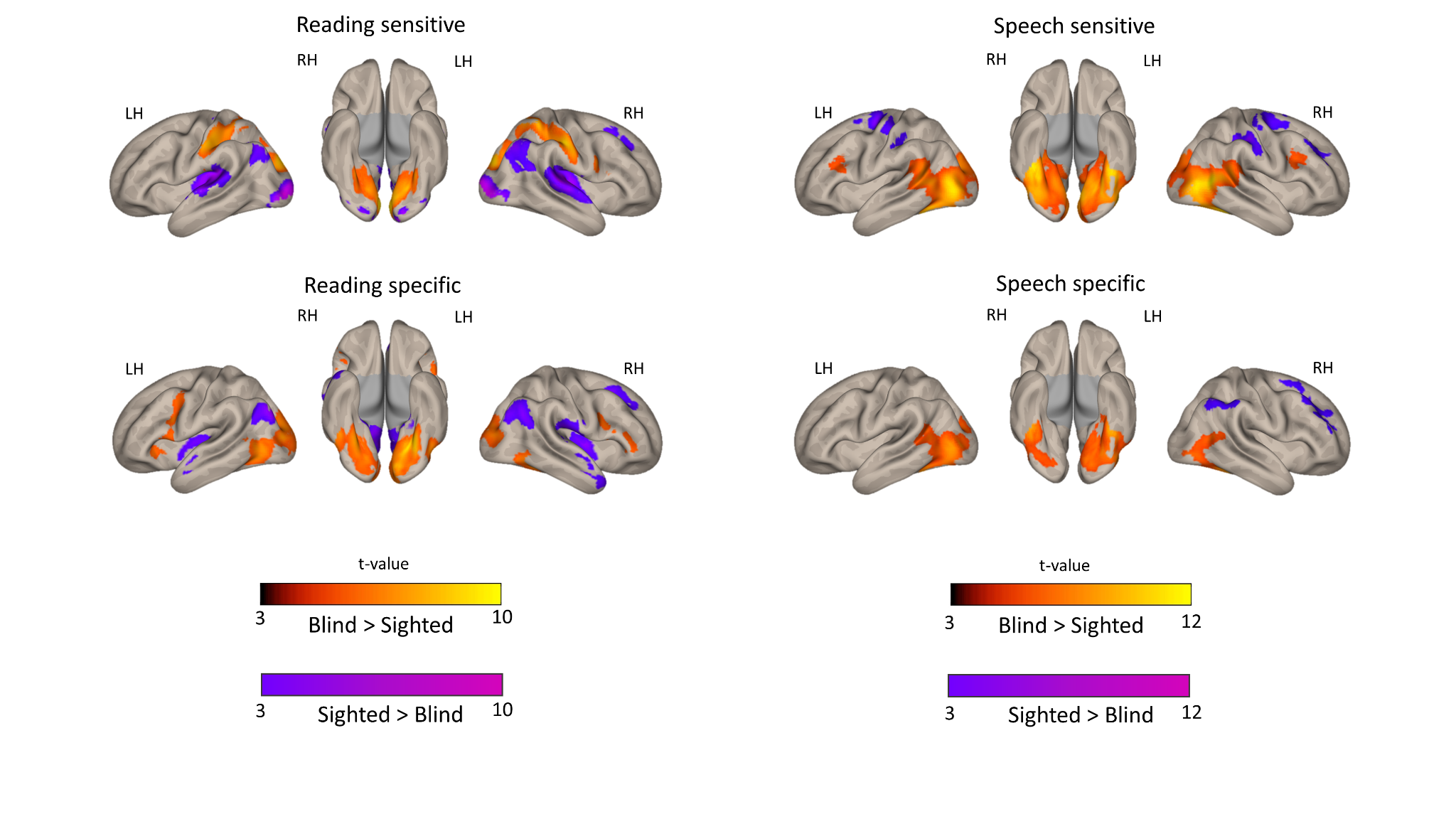


Figure S2. Group differences for the language-sensitive (words > baseline) and language-specific (words > control) contrasts.

Contrast comparing reading real words to non-linguistic control stimuli (sets of “#” symbols or six Braille dots sign) was used to delineate regions with activation specific to reading. Both blind and sighted subjects activated left vOT during reading, as well as left precentral and postcentral gyri and some parts of the primary visual cortex (Table S2, Figure S3). In the blind group, the vOT activation was bilateral and extended to large portions of the occipital cortex. The frontal activation was also bilateral and included inferior frontal gyri. When groups were compared, the blind group was shown to activate bilateral vOT, IFG and parts of precentral gyri, as well as large portions of the occipital cortices more than the sighted group (Table S2, Figure S2). The sighted group showed more activation in the bilateral temporal cortices (MTG, STG), as well as clusters in the superior medial occipitoparietal cortex and superior frontal cortex (Table S2, Figure S2).

Table S2. Group level activations and the results of the group comparison of the activations in the reading words > control contrast. The anatomical structures described according to the AAL2 using the “atlasreader” function, Hem. – hemisphere, x, y, z – peak coordinates, *t* – peak *t*-value, vol mm^3^ – cluster volume.

|  | Hem. | x | y | z | *t* | vol mm^3^ |
| --- | --- | --- | --- | --- | --- | --- |
| **Blind** |  |  |  |  |  |  |
| Middle Occipital Gyri, Lingual Gyri, Calcarine, Fusiform Gyri, Middle Temporal Gyrus, Cerebelum, Inferior Occipital Gyri, Superior Occipital Gyri, Cuneus, Inferior Temporal Gyrus, Superior Parietal Gyrus | L/R | -42 | -58 | -14 | 9.90 | 112392 |
| Inferior Frontal Gyrus (pars triangularis, opercularis, orbitalis), Precentral Gyrus, Insula, Postcentral Gyrus | L/R | -42 | -4 | 46 | 6.40 | 36520 |
| Inferior Frontal Gyrus (pars triangularis, opercularis, orbitalis), Insula, Precentral Gyrus | R | 52 | 34 | 10 | 5.22 | 13664 |
| Supplementary Motor Area | L/R | -4 | 2 | 58 | 6.18 | 9520 |
| **Sighted** |  |  |  |  |  |  |
| Inferior Occipital Gyrus, Fusiform Gyrus, Middle Occipital, Inferior Temporal Gyrus, Parahippocampal Gyrus, Cerebellum | L | -40 | -50 | -20 | 10.05 | 20368 |
| Calcarine, Precuneus, Lingual Gyrus | L/R | 16 | -48 | 6 | 5.14 | 7592 |
| Postcentral Gyrus, Precentral Gyrus | L | -48 | -6 | 50 | 4.79 | 3536 |
| **Blind > Sighted** |  |  |  |  |  |  |
| Middle Occipital Gyri, Lingual Gyri, Fusiform Gyri, Calcarine, Cerebelum, Superior Occipital Gyri, Inferior Occipital Gyri, Middle Temporal Gyrus, Cuneus, Inferior Temporal Gyri | L/R | -26 | -64 | -12 | 8.53 | 74048 |
| Precentral Gyrus, Inferior Frontal Gyrus (pars opercularis, triangularis, orbitalis), Insula | L | -36 | 6 | 22 | 4.98 | 8784 |
| Inferior Frontal Gyrus (pars triangularis) | R | 50 | 20 | -2 | 4.89 | 3456 |
| Inferior Frontal Gyrus (pars opercularis, triangularis), Precentral Gyrus | R | 46 | 10 | 26 | 4.85 | 3128 |
| Supplementary Motor Area | L/R | 10 | 20 | 48 | 4.5 | 2576 |
| **Sighted > Blind** |  |  |  |  |  |  |
| Precuneus, Middle Cingulate, Calcarine, Parahippocampal Gyri, Lingual Gyrus, Posterior Cingulate, Hippocampus, Lingual Gyrus | L/R | 16 | -50 | 8 | 6.37 | 50832 |
| Rolandic Operculum, Insula, Superior Temporal Gyrus, Heschl Gyrus, Supramarginal Gyrus, Postcentral Gyrus | R | 42 | -6 | 4 | 5.5 | 11432 |
| Angular Gyrus, Middle Occipital Gyrus, Middle Temporal Gyrus | R | 42 | -74 | 30 | 4.85 | 5344 |
| Anterior Cingulate, Superior Frontal Gyri | L/R | -2 | 44 | -6 | 4.27 | 5264 |
| Insula, Superior Temporal Gyrus, Heschl Gyrus, Middle Temporal Gyrus, Rolandic Operculum | L | -44 | -8 | 4 | 5.92 | 4736 |
| Middle Occipital Gyrus, Angular Gyrus | L | -40 | -76 | 30 | 6.06 | 3792 |
| Superior Frontal Gyrus, Middle Frontal Gyrus | R | 26 | 16 | 48 | 4.82 | 3496 |
| Middle Temporal Pole, Middle Temporal Gyrus, Superior Temporal Gyrus | R | 46 | 16 | -32 | 4.2 | 2728 |


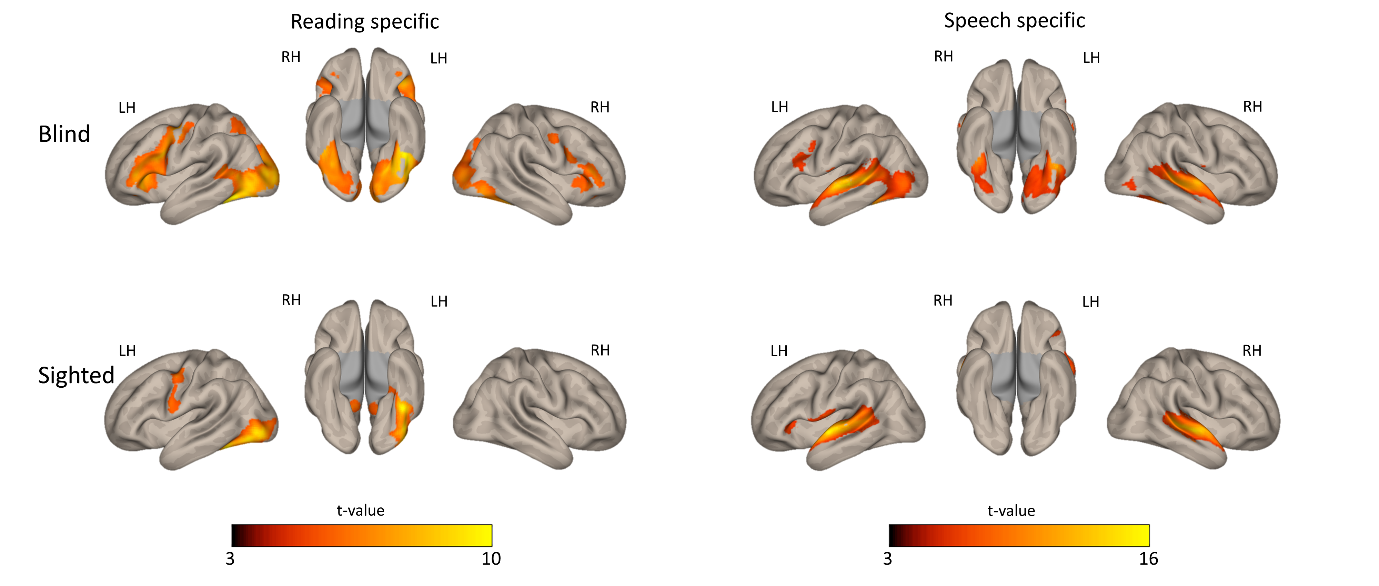


Figure S3. Group level activations for the reading words > control and speech words > control contrasts.

Speech

In the speech-sensitive contrasts (words > baseline), the blind group, apart from the bilateral temporal regions activated also by the sighted group, activated the occipital cortex with bilateral vOT and right IFG more than the sighted group (Table S3, Figure S1, S2). A contrast comparing listening to real words to non-linguistic control stimuli (vocoded speech) was used to delineate regions specifically activated by speech processing. Both groups activated bilateral superior regions (MTG, STG) and left IFG in response to speech (Table S4, Figure S3). Additionally, in the blind group, bilateral vOT activation was observed. Bilateral vOT was activated more by the blind group than by the sighted group (Table S4, Figure S2). The sighted subjects presented higher activation than the blind group in two right hemisphere clusters - in the frontal and parietal cortex (Table S4, Figure S2).

Table S3. Group level activations and the results of the group comparison of the activations in the speech words > baseline contrast. The anatomical structures described according to the AAL2 using the “atlasreader” function, Hem. – hemisphere, x, y, z – peak coordinates, *t* – peak *t*-value, vol mm^3^.

|  | Hem. | x | y | z | *t* | vol mm^3^ |
| --- | --- | --- | --- | --- | --- | --- |
| **Blind** |  |  |  |  |  |  |
| Middle Temporal Gyrus, Superior Temporal Gyrus, Inferior Frontal Gyrus (pars triangularis, opercularis, orbitalis), Lingual Gyri, Calcarine, Middle Occipital Gyrus, Fusiform Gyrus, Inferior Occipital Gyrus, Superior Temporal Pole, Cuneus, Rolandic Operculus, Supramarginal Gyrus, Insula, Precentral Gyrus, Cerebellum, Heschl Gyrus, Inferior Temporal Gyrus, Superior Occipital Gyrus, Postcentral Gyrus | L/R | -56 | -24 | 2 | 21.3 | 123128 |
| Superior Temporal Gyrus, Middle Temporal Gyrus, Fusiform Gyrus, Superior Temporal Pole, Rolandic Operculum, Insula, Cerebellum, Inferior Occipital Gyrus, Heschl Gyrus, Inferior Temporal Gyrus, Middle Occipital Gyrus, Supramarginal Gyrus, Middle Temporal Pole | R | 56 | -26 | 4 | 22.3 | 69368 |
| Inferior Frontal Gyrus (pars triangularis, opercularis), Precentral Gyrus | R | 46 | 14 | 26 | 7.31 | 10144 |
| **Sighted** |  |  |  |  |  |  |
| Superior Temporal Gyrus, Middle Temporal Gyrus, Rolandic Operculum, Superior Temporal Pole, Insula, Heschl Gyrus, Supramarginal Gyrus, Postcentral Gyrus | L | -60 | -22 | 2 | 23.2 | 50104 |
| Superior Temporal Gyrus, Middle Temporal Gyrus, Rolandic Operculum, Superior Temporal Pole, Insula, Heschl Gyrus, Supramarginal Gyrus, Postcentral Gyrus | R | 62 | -22 | 6 | 27.4 | 47904 |
| Inferior Frontal Gyrus (pars triangularis, opercularis) | L | -44 | 10 | 22 | 4.9 | 2280 |
| **Blind > Sighted** |  |  |  |  |  |  |
| Lingual Gyri, Middle Temporal Gyri, Middle Occipital Gyri, Calcarine, Fusiform Gyri, Cuneus, Inferior Occipital Gyri, Superior Temporal Gyri, Superior Occipital Gyri, Cerebellum, Inferior Temporal Gyri, Supramarginal Gyrus, Parahippocampal Gyrus | L/R | 48 | -70 | 0 | 11.3 | 170432 |
| Inferior Frontal Gyrus (pars opercularis, triangularis) | R | 38 | 12 | 28 | 4.53 | 2400 |
| Inferior Frontal Gyrus (pars triangularis) | L | -44 | 22 | 24 | 4.43 | 2080 |
| **Sighted > Blind** |  |  |  |  |  |  |
| Supplementary Motor Area, Precentral Gyri, Postcentral Gyrus, Superior Frontal Gyri, Middle Cingulate, Inferior Parietal Lobule | L/R | -4 | -6 | 54 | 6.42 | 30320 |
| Postcentral Gyrus, Supramarginal Gyrus | R | 44 | -24 | 44 | 6.29 | 6408 |
| Middle Frontal Gyrus | R | 32 | 36 | 32 | 4.45 | 2752 |

Table S4. Group level activations and the results of the group comparison of the activations in the speech words > control contrast. The anatomical structures described according to the AAL2 using the “atlasreader” function, Hem. – hemisphere, x, y, z – peak coordinates, *t* – peak *t*-value, vol mm^3^.

|  | Hem. | x | y | z | *t* | vol mm^3^ |
| --- | --- | --- | --- | --- | --- | --- |
| **Blind** |  |  |  |  |  |  |
| Middle Temporal Gyrus, Fusiform Gyrus, Superior Temporal Gyrus, Lingual Gyrus, Middle Occipital Gyrus, Inferior Occipital Gyrus, Inferior Temporal Gyrus, Superior Temporal Pole, Calcarine, Cerebellum | L | -60 | -24 | -2 | 15.24 | 47672 |
| Superior Temporal Gyrus, Middle Temporal Gyrus, Superior Temporal Pole, Middle Temporal Pole | R | 54 | -26 | 0 | 11.68 | 19832 |
| Fusiform Gyrus, Inferior Occipital Gyrus, Inferior Temporal Gyrus | R | 40 | -44 | -18 | 9.17 | 5328 |
| Inferior Frontal Gyrus (pars triangularis) | L | -50 | 26 | 20 | 4.74 | 2920 |
| **Sighted** |  |  |  |  |  |  |
| Middle Temporal Gyrus, Superior Temporal Gyrus, Superior Temporal Pole, Rolandic Operculum | L | -60 | -8 | -4 | 15.23 | 24520 |
| Superior Temporal Gyrus, Middle Temporal Gyrus, Superior Temporal Pole, Middle Temporal Pole | R | 56 | -20 | -2 | 14.82 | 21200 |
| Supplementary Motor Area | L/R | -4 | 2 | 62 | 5.3 | 2840 |
| Inferior Frontal Gyrus (pars triangularis), Insula | L | -38 | 30 | 0 | 4.9 | 1920 |
| **Blind > Sighted** |  |  |  |  |  |  |
| Middle Temporal Gyrus, Fusiform Gyrus, Middle Occipital Gyrus, Lingual Gyrus, Inferior Occipital Gyrus, Inferior Temporal Gyrus, Cerebelum, Superior Occipital Gyrus | L | -38 | -50 | -16 | 8.29 | 34720 |
| Fusiform Gyrus, Middle Temporal Gyrus, Inferior Occipital Gyrus, Inferior Temporal Gyrus, Middle Occipital Gyrus | R | 40 | -44 | -18 | 7.94 | 11760 |
| **Sighted > Blind** |  |  |  |  |  |  |
| Middle Frontal Gyrus, Superior Frontal Gyrus | R | 34 | 24 | 48 | 4.37 | 5920 |
| Inferior Parietal Lobule, Angular Gyrus, Supramarginal Gyrus | R | 46 | -56 | 44 | 4.25 | 2816 |

Reading & Speech conjunction

Lastly, speech-reading conjunction was tested in both groups. To that end, the conjunctions of the words > baseline contrasts in reading and speech processing modalities were tested. In the sighted group, the speech-reading conjunction was observed in typical perisylvian areas (bilateral MTG, STG, left IFG). In the blind group, the conjunction was found in the bilateral vOT, as well as in the left IFG (Table S5, Figure S4).

Table S5. Group-level speech-reading conjunctions. The anatomical structures described according to the AAL2 using the “atlasreader” function, Hem. – hemisphere, x, y, z – peak coordinates, *t* – peak *t*-value, vol mm^3^.

|  | Hem. | x | y | z | *t* | vol mm^3^ |
| --- | --- | --- | --- | --- | --- | --- |
| **Blind** |  |  |  |  |  |  |
| Fusiform Gyrus, Middle Occipital Gyrus, Inferior Occipital Gyrusm, Cerebellum, Lingual Gyrus, Inferior Temporal Gyrus, Middle Temporal Gyrus, Calcarine, Superior Occipital Gyrus | L | -42 | -58 | -12 | 6.77 | 18144 |
| Cerebellum, Fusiform Gyrus, Inferior Temporal Gyrus, Inferior Occipital Gyrus | R | 32 | -64 | -22 | 6.09 | 6928 |
| Inferior Frontal (pars opercularis, triangularis), Precentral Gyrus | L | -48 | 12 | 26 | 5.63 | 3736 |
| **Sighted** |  |  |  |  |  |  |
| Superior Temporal Gyrus, Middle Temporal Gyrus, Rolandic Operculum, Heschl Gyrus | L | -60 | -22 | 2 | 5.82 | 13176 |
| Superior Temporal Gyrus, Middle Temporal Gyrus | R | 52 | -34 | 4 | 5.95 | 10136 |
| Inferior Frontal Gyrus (pars triangularis, opercularis) | L | -42 | 8 | 24 | 5.04 | 2608 |


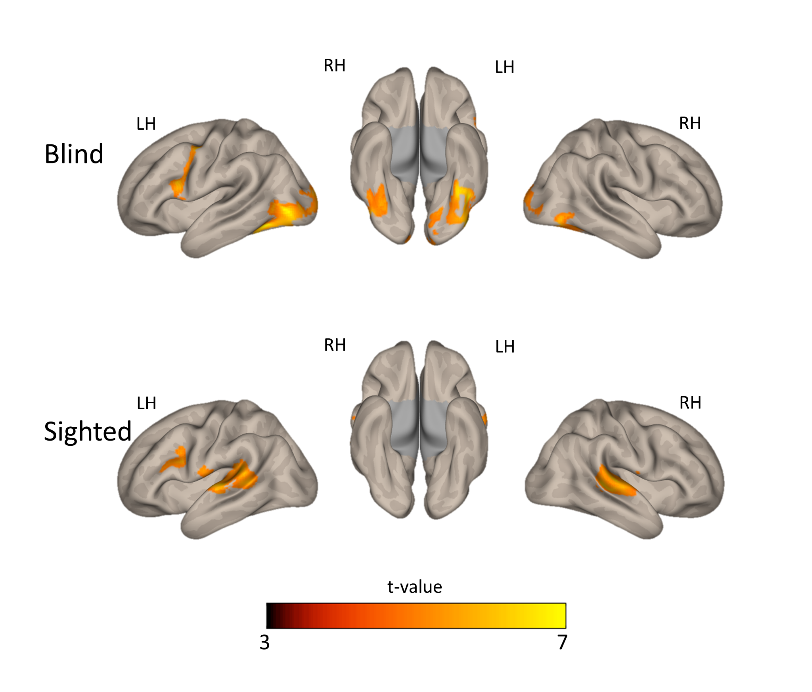


Figure S4. Speech-reading conjunction in the two groups.

Phonological task

The phonological task (rhyming > baseline contrast) induced activation of an extensive network including perisylvian (IFG, MTG, STG), parietal and occipital regions in both blind and sighted subjects (Table S6, Figure S5). The blind group activated occipital regions, including bilateral vOT to a larger extent than the sighted group (peak of activation in the left vOT region as defined by the literature ROI in -36 -48 -18). No regions were showing stronger activation in the sighted than in the blind. In the blind, the control task (control > baseline contrast) also activated the perisylvian, parietal and occipital cortex (Table S7, Figure S6). In the sighted, the occipital activation (including vOT) was not present. The significant differences between the groups were found in the bilateral occipital cortex (including vOT, peak at -44 -66 -10), and bilateral IFG, activated to a larger extent by the blind subjects.


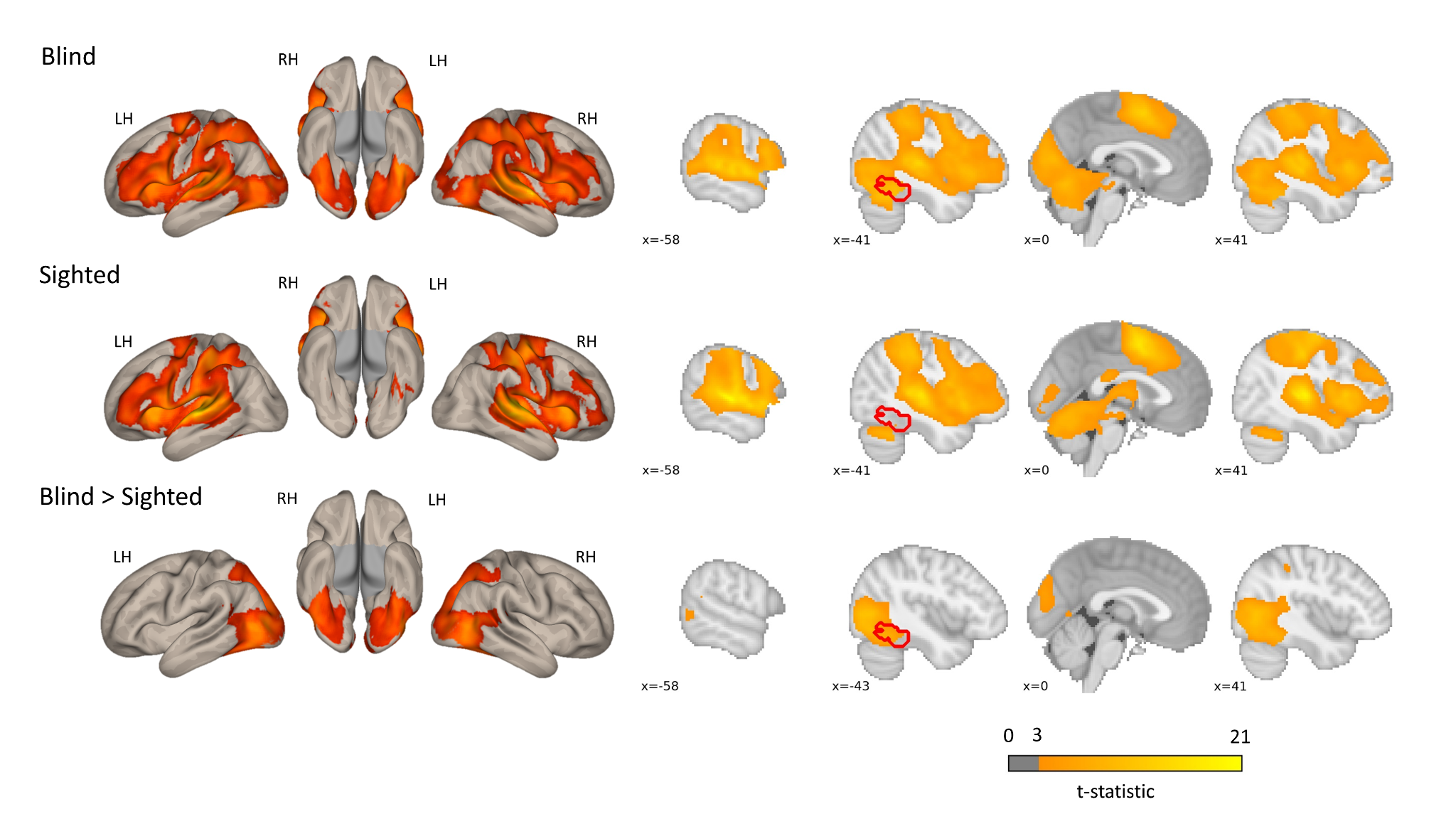


Figure S5. Group level activations from the rhyming > baseline contrast, left vOT marked in red.

Table S6. Group level activations in the rhyming > baseline contrast. The anatomical structures described according to the AAL2 using the “atlasreader” function, Hem. – hemisphere, x, y, z – peak coordinates, *t* – peak *t*-value, vol mm^3^.

|  | Hem. | x | y | z | *t* | vol mm^3^ |
| --- | --- | --- | --- | --- | --- | --- |
| **Blind** |  |  |  |  |  |  |
| Superior Temporal Gyrus, Inferior Frontal Gyrus (pars triangularis, opercularis, orbitalis), Precentral Gyrus, Middle Temporal Gyrus, Postcentral Gyrus, Middle Frontal Gyrus, Supplementary Motor Area, Cerebellum, Inferior Parietal Lobule, Middle Occipital Gyrus, Lingual Gyrus, Insula, Supramarginal Gyrus, Calcarine, Fusiform Gyrus, Superior Parietal Gyrus, Rolandic Operculum, Putamen, Superior Frontal Gyrus, Cuneus, Superior Occipital Gyrus, Thalamus, Inferior Occipital Gyrus, Middle & Anterior Cingulate, Superior Temporal Pole, Inferior Occipital Gyrus, Inferior Temporal Gyrus, Heschl Gyrus, Pallidum, Caudate, Angular Gyrus, Amygdala, Precuneus, Paracentral Lobule | R/L | 62 | -10 | 0 | 18.21 | 638928 |
| **Sighted** |  |  |  |  |  |  |
| Superior Temporal Gyrus, Postcentral Gyrus, Inferior Frontal Gyrus (pars triangularis, opercularis, orbitalis), Precentral Gyrus, Inferior Parietal Lobule, Middle Temporal Gyrus, Supplementary Motor Area, Middle Frontal Gyrus, Insula, Cerebellum, Rolandic Operculum, Supramarginal Gyrus, Putamen, Middle & Anterior Cingulate, Thalamus, Superior Frontal Gyrus, Caudate, Superior Temporal Pole, Superior Parietal Gyrus, Pallidum, Heschl Gyrus, Amygdala, Lingual Gyrus, Fusiform Gyrus, Hippocampus, Paracentral Lobule, Inferior Temporal Gyrus | R/L | 56 | -18 | 4 | 21.23 | 545624 |
| Calcarine, Lingual Gyrus | R/L | -12 | -72 | 8 | 5.66 | 7664 |
| **Blind > Sighted** |  |  |  |  |  |  |
| Middle Occipital Gyrus, Fusiform Gyrus, Middle Temporal Gyrus, Superior Occipital Gyrus, Cuneus, Inferior Occipital Gyrus, Lingual Gyrus, Superior Parietal Gyrus, Inferior Temporal Gyrus, Calcarine, Precuneus, Superior Temporal Gyrus, Cerebellum, Inferior Parietal Lobule, Angular Gyrus, Postcentral Gyrus | R/L | -38 | -70 | 4 | 11.33 | 150808 |


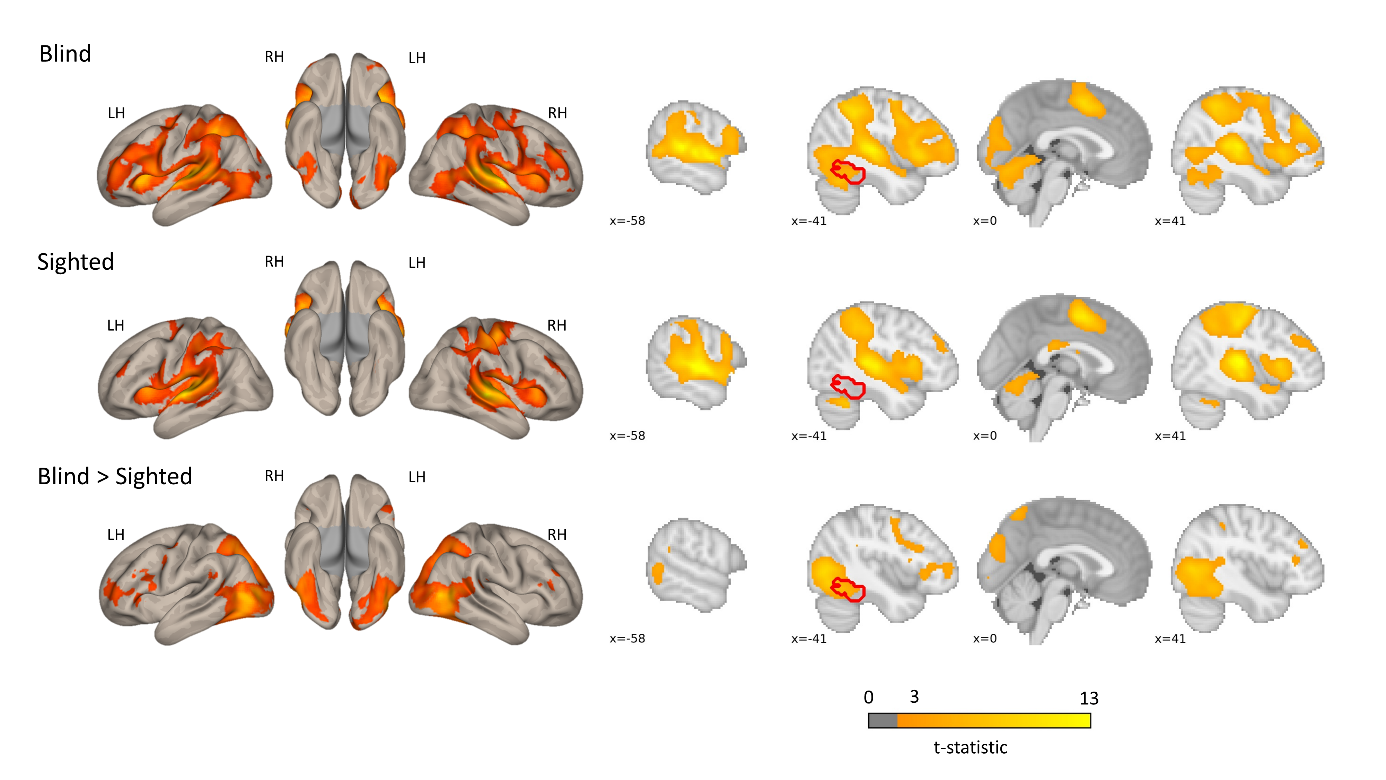


Figure S6. Group level activations and the group comparison of the activations from the control > baseline contrast, left vOT marked in red.

Table S7. Group level activations and the results of the group comparison of the activations in the control > baseline contrast. The anatomical structures described according to the AAL2 using the “atlasreader” function, Hem. – hemisphere, x, y, z – peak coordinates, *t* – peak *t*-value, vol mm^3^.

|  | Hem. | x | y | z | *t* | vol mm^3^ |
| --- | --- | --- | --- | --- | --- | --- |
| **Blind** |  |  |  |  |  |  |
| Superior Temporal Gyri, Inferior Frontal Gyri (pars triangularis, opercularis), Inferior Parietal Lobule, Middle Temporal Gyri, Middle Frontal Gyri, Precentral Gyri, Cerebellum, Supramarginal Gyri, Insula, Supplementary Motor Area, Postcentral Gyri, Superior Parietal Gyrus, Rolandic Operculum, Middle Occipital Gyri, Calcarine, Putamen, Cuneus, Thalamus, Fusiform Gyri, Superior Frontal Gyrus, Middle Cingulate, Inferior Occipital Gyri, Superior Occipital Gyri, Inferior Temporal Gyri, Superior Temporal Pole, Heschl Gyri, Angular Gyri, Precuneus, Pallidum, Lingual Gyri, Caudate | R/L | 62 | -14 | 2 | 16.35 | 400272 |
| **Sighted** |  |  |  |  |  |  |
| Superior Temporal Gyri, Inferior Parietal Lobule, Postcentral Gyri, Middle Temporal Gyri, Insula, Supplementary Motor Area, Precentral Gyri, Rolandic Operculum, Supramarginal Gyri, Putamen, Inferior Frontal Gyri (pars opercularis, triangularis, orbitalis), Thalamus, Middle Cingulate, Superior Temporal Pole, Superior Frontal Gyri, Caudate, Pallidum, Superior Parietal Gyri, Heschl Gyri, Caudate, Angular Gyri, Middle Frontal Gyri, Anterior Cingulate, Middle Occipital Gyrus | R/L | 58 | -16 | 2 | 17.00 | 268864 |
| Cerebellum | L | -24 | -50 | -28 | 11.84 | 18184 |
| Cerebellum | R | 28 | -56 | -26 | 7.05 | 5144 |
| Middle Frontal Gyrus, Inferior Frontal Gyrus (pars triangularis), Superior Frontal Gyrus | R | 36 | 42 | 26 | 6.23 | 4624 |
| Middle Frontal Gyrus | L | -38 | 40 | 32 | 4.76 | 2912 |
| **Blind > Sighted** |  |  |  |  |  |  |
| Middle Occipital Gyri, Middle Temporal Gyri, Superior Occipital Gyri, Superior Parietal Gyri, Cuneus, Fusiform Gyri, Inferior Occipital Gyri, Inferior Temporal Gyri, Superior Temporal Gyri, Precuneus, Inferior Parietal Lobule, Calcarine, Lingual Gyri, Angular Gyrus, Cerebellum, Supramarginal Gyri, Rolandic Operculum, Postcentral Gyrus | R/L | 50 | -66 | 2 | 10.26 | 131920 |
| Middle Frontal Gyrus, Inferior Frontal Gyrus (pars triangularis, orbitalis), Superior Frontal Gyrus, Insula | L | -34 | 52 | 12 | 4.52 | 8328 |
| Precentral Gyrus, Inferior Frontal Gyrus (pars triangularis, opercularis), Middle Frontal Gyrus | L | -48 | 10 | 32 | 4.46 | 4848 |

Previous literature has shown that in the sighted, vOT activation during speech stimuli presentation is task-dependent and characterized by a deactivation when the task does not require access to orthographic representations (Yoncheva et al. 2010, Ludersdorfer et al. 2016). Because of that, deactivation during both tasks was also analysed here. In both the rhyming task (baseline > rhyming contrast) and control task (baseline > control contrast) both blind and sighted subjects showed deactivation mainly in regions that are a part of the default mode network (anterior, middle and posterior cingulate, angular gyrus, precuneus, medial frontal cortex). However, in the sighted group, occipital regions were largely deactivated too. During the control task, the deactivation included bilateral vOT regions (peak at -32 -10 -16, Table S8, Figure S7).


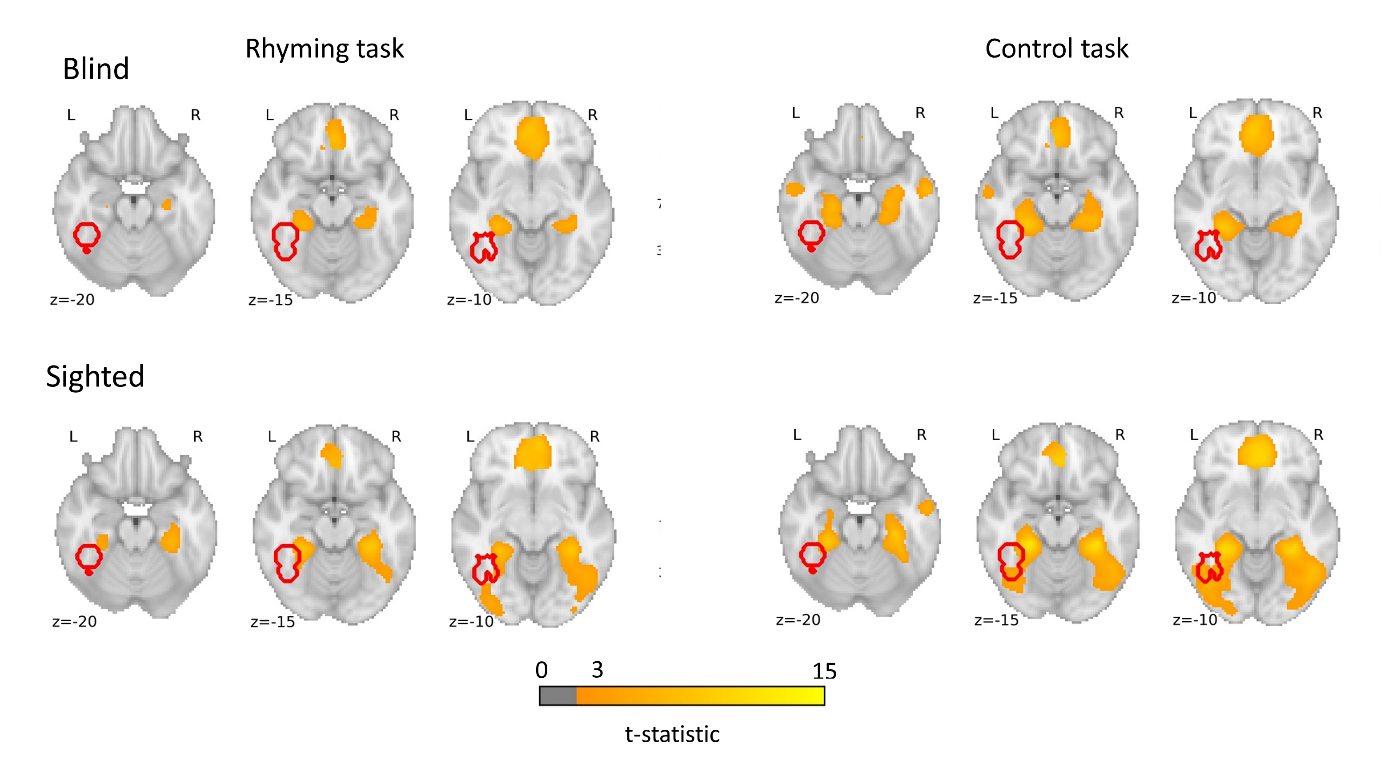


Figure S7. Deactivations in the phonological (baseline > rhyming contrast) and control (baseline > control contrast) tasks, left vOT marked in red.

Table S8. Group level deactivations in the phonological (baseline > rhyming contrast) and control task (baseline > control contrast). The anatomical structures described according to the AAL2 using the “atlasreader” function, Hem. – hemisphere, x, y, z – peak coordinates, *t* – peak *t*-value, vol mm^3^.

|  | Hem. | x | y | z | *t* | vol mm^3^ |
| --- | --- | --- | --- | --- | --- | --- |
| **Rhyming** |  |  |  |  |  |  |
| **Blind** |  |  |  |  |  |  |
| Precuneus, Middle Cingulate, Posterior Cingulate, Calcarine, Cuneus, Lingual Gyri, Hippocampus | R/L | -10 | -56 | 18 | 9.91 | 40704 |
| Superior Frontal Gyrus, Anterior Cingulate, Olfactory cortex | R/L | -8 | 44 | -6 | 8.70 | 28048 |
| Angular Gyrus, Middle Occipital Gyrus, Middle Temporal Gyrus | R | 42 | -74 | 32 | 11.38 | 9368 |
| Angular Gyrus, Middle Occipital Gyrus, Inferior Parietal Lobule | L | -42 | -72 | 34 | 11.63 | 8952 |
| Superior Frontal Gyrus, Middle Frontal Gyrus | R | 24 | 30 | 44 | 7.68 | 6712 |
| Superior Frontal Gyrus, Middle Frontal Gyrus | L | -24 | 32 | 46 | 7.1 | 4760 |
| Parahippocampal Gyrus, Hippocampus, Fusiform Gyrus | L | -30 | -36 | -10 | 6.7 | 3680 |
| Parahippocampal Gyrus, Hippocampus | R | 32 | -36 | -10 | 5.38 | 3288 |
| **Sighted** |  |  |  |  |  |  |
| Middle Occipital Gyri, Precuneus, Middle Temporal Gyri, Superior Occipital Gyri, Angular Gyri, Middle Cingulate, Fusiform Gyrus, Cuneus, Inferior Occipital Gyri | R/L | -42 | -74 | 28 | 14.75 | 158592 |
| Superior Frontal Gyri, Anterior Cingulate, Middle Frontal Gyrus | R/L | 4 | 54 | -6 | 10.27 | 43080 |
| Superior Frontal Gyrus, Middle Frontal Gyrus | R | 24 | 24 | 44 | 8.64 | 7456 |
| Fusiform Gyrus, Parahippocampal Gyrus | L | -30 | -38 | -14 | 8.41 | 5008 |
| **Control** |  |  |  |  |  |  |
| **Blind** |  |  |  |  |  |  |
| Precuneus, Middle & Posterior Cingulate, Parahippocampal Gyrus, Paracentral Lobule, Hippocampus Calcarine, Fusiform, Lingual Gyrus, Cuneus, Cerebellum | R/L | 10 | -52 | 16 | 9.38 | 76112 |
| Superior Frontal Gyrus, Anterior Cingulate, Middle Frontal Gyrus | L | -4 | 44 | -8 | 8.85 | 42984 |
| Middle Occipital Gyrus, Angular Gyrus, Middle Temporal Gyrus | L | -40 | -76 | 32 | 11.07 | 10904 |
| Superior Frontal Gyrus, Middle Frontal Gyrus | R/L | 26 | 30 | 46 | 7.79 | 10448 |
| Middle Occipital Gyrus, Angular Gyrus, Middle Temporal Gyrus | R/L | 44 | -74 | 26 | 9.11 | 9680 |
| Middle Temporal Gyrus, Middle & Superior Temporal Pole | R/L | 44 | 14 | -32 | 8.83 | 6080 |
| Middle Temporal Gyrus, Middle Temporal Pole, Inferior Temporal Gyrus | L | -42 | 10 | -34 | 5.43 | 3136 |
| **Sighted** |  |  |  |  |  |  |
| Middle Occipital Gyrus, Precuneus, Middle Occipital Gyrus, Middle Temporal Gyrus, Superior Occipital Gyrus, Fusiform Gyrus, Middle & Posterior Cingulate, Inferior Occipital Gyrus, Inferior Temporal Gyrus, Angular Gyrus, Cuneus, Parahippocampal Gyrus, Calcarine, Paracentral Lobule, Lingual Gyrus, Superior Parietal Gyrus, Postcentral Gyrus, Hippocampus, Inferior Parietal Lobule, Postcentral Gyrus | R/L | 46 | -72 | 22 | 13.3 | 206504 |
| Superior Frontal Gyrus, Anterior Cingulate, Middle Frontal Gyrus | L | -4 | 38 | -6 | 11.03 | 58016 |
| Superior Frontal Gyrus, Middle Frontal | R | 24 | 28 | 46 | 7.54 | 6832 |
| Middle Temporal Gyrus, Middle Temporal Pole | R | 40 | 18 | -34 | 5.73 | 3856 |
| Postcentral Gyrus, Precentral Gyrus | R | 46 | -8 | 34 | 6.38 | 3232 |
| Postcentral Gyrus, Precentral Gyrus | L | -48 | -10 | 32 | 5.78 | 2976 |

Correlations with reading level, phonological skills level and age

Literature-based and individual language-sensitive left vOT ROI

We tested the correlation between the reading level, age, phonological skills and the mean contrast estimates from the three tasks: rhyming and control phonological tasks and the auditory non-linguistic control condition from the localizer task, to test whether the correlations were specific to language processing or rather connected in general to auditory processing. The mean contrast estimates were extracted from the literature-based and the individual vOT ROIs. The reading level was measured using a reading test created for the purposes of the study as no standardised reading-level measures exist for the Polish blind population. Subjects were presented with lists of words of increasing length and asked to read them out loud as fast as they could. Accuracy and speed were checked. The mean number of words read correctly per minute was treated as an indicator of reading skill. Phonological skills were measured with a vowel replacement task adopted from (Szczerbiński, 2001). Participants were asked to replace the vowel /a/ with /u/ in words pronounced by the experimenter. Participants were presented with three training items first and then with test items. The first 8 test items each contained only one vowel /a/ (e.g. /rak/ [crayfish]), and the other 8 contained two vowels /a/ each (e.g. /fala/ [wave]). Time and accuracy were measured in both parts of the test. The outcome variable was the accuracy/time ratio. The correlation of the phonological skills level with age was tested within the blind and sighted groups to explore changes in this capacity with development. In both blind (*rho* = 0.53, *p* < 0.001) and sighted (*rho* = 0.60, *p* < 0.001), the correlation between the phonological task score and age was positive and significant.

The non-parametric Spearman’s *rho* coefficient was used as the data distribution for the rhyming task-related contrast estimates extracted from the literature-based ROI turned out not to be normal. Bonferroni-Holm correction for the multiple comparisons was used.

The results of the analyses were independent of the ROI definition method. The correlation between reading level and activation during the rhyming was significant only in the blind subjects. All of the other correlations (with the control task and for vocoded speech processing in the blind, as well as all of the correlations in the sighted subjects) were insignificant. The correlations with the vowel replacement task were also significant only in the blind group. This phonological task score correlated with the activity during both rhyming and control tasks. The exact values of the correlation coefficients are presented in Table S10.

As reading level was highly correlated with age (Blind: *rho* = 0.65, *p* < 0.001, Sighted: *rho* = 0.55, *p* < 0.001) it was not possible to disentangle the influence of both factors on the left vOT activation. The pattern of correlations was similar for age and reading level (Table S9). Partial correlations controlling for the second factor were insignificant for both age and reading level (Table S10). The only exception was the correlation with reading level controlled for age and the left vOT activation in the individual ROIs in the blind group, *rho* = 0.35, *p* = 0.042.

Table S9. Correlations between the reading level, vowel replacement task score, age and mean contrast estimates for rhyming and control tasks extracted from literature-based and individual ROIs. *P*-values corrected using the Bonferroni-Holm correction presented.

|  |  |  | Literature ROI | | Individual ROIs | |
| --- | --- | --- | --- | --- | --- | --- |
|  |  |  | Blind | Sighted | Blind | Sighted |
| words per minute | rhyming task | *rho* | **0.41** | 0.28 | **0.46** | 0.28 |
|  |  | *p* | **0.009** | 0.132 | **0.003** | 0.120 |
|  | control task | *rho* | 0.15 | 0.09 | 0.28 | 0.16 |
|  |  | *p* | 0.578 | 0.511 | 0.092 | 0.472 |
|  | vocoded speech | *rho* | 0.13 | -0.27 | -0.02 | -0.08 |
|  |  | *p* | 0.353 | 0.094 | 0.916 | 0.524 |
| age | rhyming task | *rho* | **0.48** | 0.23 | 0.31 | 0.14 |
|  |  | *p* | **0.002** | 0.178 | 0.081 | 0.933 |
|  | control task | *rho* | 0.20 | 0.01 | 0.25 | 0.03 |
|  |  | *p* | 0.156 | 0.961 | 0.152 | 0.855 |
|  | vocoded speech | *rho* | 0.31 | -0.06 | 0.10 | -0.13 |
|  |  | *p* | 0.060 | 1.000 | 0.474 | 0.688 |
| vowel replacement | rhyming task | *rho* | **0.45** | 0.23 | **0.37** | 0.20 |
|  |  | *p* | **0.004** | 0.315 | **0.028** | 0.310 |
|  | control task | *rho* | **0.37** | 0.01 | **0.35** | -0.04 |
|  |  | *p* | **0.026** | 0.961 | **0.029** | 0.763 |
|  | vocoded speech | *rho* | -0.19 | -0.11 | -0.18 | -0.26 |
|  |  | *p* | 0.198 | 0.895 | 0.217 | 0.200 |

Table S10. Partial correlations with reading level and age in the literature-based and individual vOT ROIs. *P*-values corrected using the Bonferroni-Holm correction presented.

|  |  | Reading level controlled for age | | | | Age controlled for reading level | | | |
| --- | --- | --- | --- | --- | --- | --- | --- | --- | --- |
|  |  | Literature ROI | | Individual ROIs | | Literature ROI | | Individual ROIs | |
|  |  | Blind | Sighted | Blind | Sighted | Blind | Sighted | Blind | Sighted |
| rhyming task | *rho* | 0.16 | 0.18 | **0.35** | 0.25 | 0.29 | 0.10 | 0.02 | -0.02 |
|  | *p* | 0.828 | 0.390 | **0.042** | 0.225 | 0.088 | 0.910 | 1.000 | 1.000 |
| control task | *rho* | 0.03 | 0.10 | 0.16 | 0.18 | 0.14 | -0.05 | 0.10 | -0.07 |
|  | *p* | 0.856 | 0.460 | 0.532 | 0.398 | 0.338 | 0.712 | 0.514 | 0.580 |
| vocoded speech | *rho* | -0.09 | -0.29 | -0.11 | -0.02 | 0.29 | 0.11 | 0.15 | -0.10 |
|  | *p* | 1.000 | 0.114 | 0.455 | 0.911 | 0.120 | 1.000 | 0.918 | 1.000 |

Reading and speech selective left vOT

The correlations with reading level, phonological task score and age were tested in the reading- and speech-selective ROIs as well. The Spearman’s *rho* coefficient was also used. Most of the correlations were not significant with one exception (see Table S11). The correlation between rhyming task activation with the vowel replacement score was significant in the reading-selective ROI in the blind group.

Table S11. Correlations between the reading level, vowel replacement task score, and mean contrast estimates for rhyming and control tasks extracted from individual reading- and speech-specific ROIs. *P*-values corrected using the Bonferroni-Holm correction presented.

|  |  |  | Speech-specific ROI | | Reading-specific ROI | |
| --- | --- | --- | --- | --- | --- | --- |
|  |  |  | Blind | Sighted | Blind | Sighted |
| words per minute | rhyming task | *rho* | 0.16 | 0.19 | 0.33 | 0.19 |
|  |  | *p* | 0.757 | 0.504 | 0.065 | 0.317 |
|  | control task | *rho* | 0.01 | -0.02 | 0.00 | -0.03 |
|  |  | *p* | 0.966 | 0.874 | 0.977 | 0.854 |
|  | vocoded speech | *rho* | 0.06 | 0.14 | 0.05 | 0.23 |
|  |  | *p* | 1.000 | 0.646 | 1.000 | 0.284 |
| age | rhyming task | *rho* | 0.22 | 0.18 | 0.24 | 0.04 |
|  |  | *p* | 0.393 | 0.546 | 0.265 | 0.751 |
|  | control task | *rho* | 0.15 | -0.07 | 0.09 | -0.26 |
|  |  | *p* | 0.576 | 1.000 | 0.546 | 0.165 |
|  | vocoded speech | *rho* | 0.11 | -0.01 | 0.19 | 0.12 |
|  |  | *p* | 0.449 | 0.952 | 0.368 | 0.800 |
| vowel replacement | rhyming task | *rho* | 0.15 | 0.21 | **0.36** | -0.05 |
|  |  | *p* | 0.608 | 0.411 | **0.039** | 0.728 |
|  | control task | *rho* | 0.13 | -0.07 | 0.20 | -0.18 |
|  |  | *p* | 0.366 | 0.612 | 0.336 | 0.603 |
|  | vocoded speech | *rho* | -0.19 | -0.12 | -0.10 | -0.08 |
|  |  | *p* | 0.618 | 0.764 | 0.486 | 1.000 |

Analyses without the early blind participants

As the onset of blindness can alter the functional organization of the brain (Burton et al., 2002, Bedny et al., 2012), we have conducted the analyses with (*N* = 50) and without (*N* = 41) the nine subjects that lost their sight after birth. As the differences were minor, we reported only the whole-sample results in the main text.

Whole-brain results

Localizer

The activations for the reading-specific contrast (reading words > control) were present in bilateral vOT extending to the occipital cortex, bilateral IFG and pre/postcentral gyri as well as in SMA. Speech processing (speech words > control) invoked activation in the bilateral auditory (MTG, STG) cortex and vOT. The cluster in the left IFG observed on the total sample was present but did not survive cluster-level correction. The speech-reading conjunction was present in the bilateral vOT. Again, the left IFG cluster observed in the total sample was present but did not survive cluster-level correction.

When it comes to group differences, during reading congenitally blind subjects activated bilateral vOT extending to the occipital cortex and left IFG more than the sighted subjects. Clusters in the right IFG and SMA that were present in the comparison of the total blind sample and the sighted groups were present but did not survive cluster-level correction. The activations higher for the sighted group than for the congenitally blind group remained the same as for the complete sample. For speech processing, the group differences were practically the same as for the complete sample. The only difference was the significance of the right IPL cluster in the sighted > congenitally blind comparison, which did not survive the cluster-level correction.

Phonological task

The activations during phonological processing (rhyming > baseline) were almost identical for the congenitally blind group and the total sample. Congenitally blind subjects activated the occipital cortex to a larger extent than the sighted subjects Sighted subjects presented higher activation in the bilateral middle cingulate than the congenitally blind group. Activations during the control task were also very similar in the congenitally and the total blind sample, as were the regions of the group differences from the sighted group. The regions of significant deactivation during both the rhyming and control task were also very similar to the complete sample.

The rhyming > control contrast again invoked very similar, however a little bit less extensive activations in the congenitally blind group and the total sample. The significant difference between the sighted and congenitally blind subjects for this contrast was again present in the left vOT on the lenient statistical threshold (*p* < 0.001, cluster extent = 50 voxels).

ROI analyses

Group and condition effects in the left vOT ROIs

For the literature-based ROI the robust two-way mixed ANOVA gave a significant main effect of group (*F*(1, 35.49) = 39.90, *p* < 0.001), main effect of condition (*F*(1, 38.58) = 44.62, *p* < 0.001) and a significant group x condition interaction (*F*(1, 38.58) = 5.75, *p* = 0.021). The results were thus very similar to the results of the whole group, with a difference in the significance of the interaction. The correlations with the reading skills were a little bit weaker than in the whole sample and the correlation with the rhyming task activation was only marginally significant (*rho* = 0.34, *p* = 0.096).

When the individual ROIs were considered, the main effect of group (*F*(1, 36.94) = 47.89, *p* < 0.001) and condition (*F*(1, 46.86) = 36.97, *p* < 0.001) were observed. As was the case in the whole sample, the group x condition interaction was insignificant (*F*(1, 46.86) = 2.36, *p* = 0.131). There was a significant correlation between the rhyming task activation and reading skills (*rho* = 0.43, *p* = 0.018) but the correlation with the control task activation was insignificant (*rho* = 0.27, *p* = 0.168). The correlation between reading and non-linguistic auditory stimuli processing (vocoded speech), remained insignificant (*rho* = 0.00, *p* = 0.983).

Comparison to the other language-network ROIs

The results of the three-way mixed ANOVA conducted with only congenitally blind subjects were the same as for the analysis on the complete sample. There was main effect of group (χ^2^(1) = 9.30, *p* = 0.002), ROI (χ^2^(3) = 526.58, *p* < 0.001) and condition (χ^2^(1) = 26.23, *p* < 0.001), as well as significant group x ROI interaction (χ^2^(3) = 45.66, *p* < 0.001). The condition x group, condition x ROI and condition x ROI x group interactions were not significant.

Post-hoc tests have shown that the activations for rhyming were higher than for the control task in both groups, for the STG and V1, and in the case of the sighted subjects vOT ROIs (Blind: V1: *p* = 0.012, STG: *p* = 0.019; Sighted: V1: *p* = 0.012, STG: *p* = 0.019, vOT: *p* = 0.040), but this difference became insignificant for Broca’s area and in case of the blind subjects the vOT ROIs (Blind: vOT: *p* = 0.128, Broca’s area: *p* = 0.077, Sighted: Broca’s area: *p* = 0.077).

Group by ROI interaction can be interpreted as stemming from the fact that in the occipital ROIs (Rhyming: V1: p = 0.050, vOT p < 0.001, Control: V1: p = 0.050, vOT p < 0.001), for both conditions, activation was higher in the blind group than in the sighted group and the differences between the groups were not significant for the Broca’s area (Rhyming: p = 0.844, Control p = 0.844) and STG ROI (Rhyming: p = 1.000, Control p = 1.000). In the blind group, for both conditions, STG ROI activation was higher than the three other ROIs (p-values of all comparisons < 0.001) and the differences between the Broca’s area, V1 and vOT were insignificant. In the sighted group on the other hand, for both conditions, not only did STG ROI have higher activation than the three other ROIs (p-values of all comparisons < 0.001) but also the Broca’s area had higher activation than the vOT ROI (*p* < 0.001). The differences between the vOT and V1, as well as V1 and Broca’s area, were not significant.

Analyses on adult participants.

In order to test whether the large age range in our participants' sample influenced the observed effects of group (Blind vs Sighted) and condition (rhyming vs control task) the analyses were conducted on the restricted group of adult participants (older than 18 years old).

Literature-based and individual language-sensitive left vOT ROIs

The results within adult group were similar to the ones observed when the whole age-range was considered. In the literature-based ROI, there was a significant main effect of group (*F*(1, 20.58) = 34.66, *p* < 0.001) and condition (*F*(1, 23.15) = 46.71, *p* < 0.001). The group by condition interaction was insignificant (*F*(1, 23.15) = 4.11, *p* = 0.054).

The results were also the same when individual ROIs were analysed. The main effects of group (*F*(1, 19.33) = 32.63, *p* < 0.001) and condition (*F*(1, 25.43) = 26.92, *p* < 0.001) were significant. The group by condition interaction was not significant (*F*(1, 25.43) = 3.06, *p* = 0.092).

Reading and speech selective left vOT

In the adult participants the effects were independent of the ROI definition and similar to the results presented in the main text. There was a significant main effect of group (reading: *F*(1, 22.52) = 16.41, *p* = 0.001, speech: *F*(1, 23.68) = 8.12, *p* = 0.009) and condition (reading: *F*(1, 26.74) = 16.91, *p* < 0.001, speech: *F*(1, 28.59) = 12.19, *p* = 0.002). The group by condition interaction was insignificant (reading: *F*(1, 26.74) = 0.04, *p* = 0.836, speech: *F*(1, 28.59) = 0.18, *p* = 0.670).

Comparison to the other language-network ROIs

In the adult participants, there was a significant effect of group (F(1, 47) = 16.80, *p* < 0.001), condition (F(1, 47) = 35.67, *p* < 0.001) and ROI (F(3, 141) = 89.75, *p* < 0.001), as well as group by ROI interaction (F(3, 141) = 6.25 *p* < 0.001). The condition by ROI (F(3, 141) = 0.37, *p* = 0.777), group by condition (F(1, 47) = 1,28 *p* = 0.263,) and group by condition by ROI (F(3, 141) = 2.23, *p* = 0.087) interactions were insignificant. Post-hoc tests have shown that the activations for rhyming were higher than for the control task in the vOT ROI in the blind (vOT: *p* = 0.013, STG: *p* = 0.101, V1: *p* = 0.991, Broca’s: *p* = 0.192) and in the STG (*p* < 0.001) and Broca’s area (*p* = 0.002) in the sighted adults (vOT: *p* = 0.139, V1: *p* = 0.068). The blind group had higher activity for both rhyming and control conditions in the occipital ROIs (rhyming: vOT *p* < 0.001, V1 *p* = 0.091, control: vOT *p* = 0.005, V1 *p* = 0.006) but not in the STG and Broca’s ROIs (rhyming: STG *p* = 1.000, Broca’s *p* = 0.773; control: STG *p* = 1.000, Broca’s *p* = 0.345). In the blind group, for both conditions, STG ROI presented higher activation than all of the other ROIs (all *p* < 0.002) but there were no differences between the V1, vOT and Broca’s ROIs. In the sighted still STG ROI had higher activation than other ROIs for both conditions (all *p* < 0.001). However, Broca’s area ROI presented higher activity than vOT for both conditions (rhyming: *p* < 0.001, control *p* = 0.018). There were no significant differences between vOT and V1 or V1 and Broca’s area (lower *p* = 0.114).
